# HIV-1 sequences in lentiviral vector genomes can be substantially reduced without compromising transduction efficiency

**DOI:** 10.1038/s41598-021-91309-w

**Published:** 2021-06-08

**Authors:** Helin Sertkaya, Mattia Ficarelli, Nathan P. Sweeney, Hannah Parker, Conrad A. Vink, Chad M. Swanson

**Affiliations:** 1 Department of Infectious Diseases, King’s College London, London, SE1 9RT UK; 2 Cell & Gene Therapy Platform, Medicinal Science and Technology, GSK, Stevenage, SG1 2NY UK

**Keywords:** Alternative splicing, Genetic vectors, Retrovirus, Transcriptomics

## Abstract

Many lentiviral vectors used for gene therapy are derived from HIV-1. An optimal vector genome would include only the viral sequences required for transduction efficiency and gene expression to minimize the amount of foreign sequence inserted into a patient’s genome. However, it remains unclear whether all of the HIV-1 sequence in vector genomes is essential. To determine which viral sequences are required, we performed a systematic deletion analysis, which showed that most of the *gag* region and over 50% of the *env* region could be deleted. Because the splicing profile for lentiviral vectors is poorly characterized, we used long-read sequencing to determine canonical and cryptic splice site usage. Deleting specific regions of *env* sequence reduced the number of splicing events per transcript and increased the proportion of unspliced genomes. Finally, combining a large deletion in *gag* with repositioning the Rev-response element downstream of the 3’ R to prevent its reverse transcription showed that 1201 nucleotides of HIV-1 sequence can be removed from the integrated vector genome without substantially compromising transduction efficiency. Overall, this allows the creation of lentiviral vector genomes that contain minimal HIV-1 sequence, which could improve safety and transfer less viral sequence into a patient’s DNA.

## Introduction

Retroviral vectors are used for a wide variety of cellular therapies^1^. While they were originally based on gammaretroviruses, human immunodeficiency virus type 1 (HIV-1) has been adapted to produce lentiviral vectors^2^. Lentiviral vectors are particularly promising gene therapy tools due to their ability to facilitate efficient transduction and long-term stable transgene expression in both dividing and non-dividing cells^2^. They also have lower risk of genotoxicity due to insertional mutagenesis than gammaretroviral vectors. Lentiviral vectors are currently used for treatments that involve gene transfer into hematopoietic stem cells and lymphocytes. One example is Kymriah, an autologous chimeric antigen receptor (CAR) T cell immunotherapy that uses a HIV-1 based lentiviral vector to treat acute lymphoblastic leukemia^3^.

HIV-1 contains nine open reading frames: the structural proteins Gag, Pol and Env, the accessory proteins Vif, Vpr, Vpu and Nef that counteract innate and adaptive immunity and the regulatory proteins Tat and Rev that control viral gene expression^4–6^. In addition, the HIV-1 genome contains many linear or structural *cis*-acting RNA elements that regulate reverse transcription, RNA transcription, pre-mRNA splicing, intron-containing RNA nuclear export, translation and genomic RNA packaging^6,7^. The first generation lentiviral vector system used three plasmids to express the viral proteins required to make infectious particles and the genome^8^. The plasmid encoding the vector genome contained all of the signals necessary for its transcription, dimerization, encapsidation, reverse transcription and integration. It also had an internal promoter to express the transgene of interest. The second plasmid was the packaging vector containing intact reading frames for all of the HIV-1 proteins except Env to express Gag and Gag-Pol as well as the accessory and regulatory proteins^8^. The third plasmid expressed a viral protein to mediate entry into the target cell, often the vesicular stomatitis virus glycoprotein (VSV-G)^8^.

Since their initial development, considerable progress has been made in lentiviral vector constructs to reduce the number of viral proteins expressed by the packaging plasmids and the amount of viral sequence in the vector genome. Second generation packaging constructs eliminated Vif, Vpr, Vpu and Nef expression while maintaining Gag, Gag-Pol, Tat and Rev^9^. In addition, the woodchuck hepatitis virus post-transcriptional regulatory element (WPRE) was introduced at the 3’ end of the vector genome^10^. While the function of this element remains unclear, it may improve vector titre and/or promote transgene expression by promoting RNA nuclear export or stability^10–16^. Third generation lentiviral vector systems further reduced the amount of viral sequence in the packaging and genome plasmids^17^. The requirement for Tat was eliminated by incorporating a constitutively active promoter in place of the HIV-1 5’ U3 in the vector genome plasmid. Because Rev is required for Gag-Pol expression and vector titre, this protein was expressed *in trans* allowing a packaging construct containing only the *gag* and *pol* genes plus the *cis*-acting Rev-response element (RRE). These modifications reduced biosafety concerns, including the potential to produce replication-competent lentivirus (RCL)^17,18^. If recombination occurred between the packaging and genome constructs, RCL particles could theoretically be produced and spread beyond the intended target cells. Therefore, the third generation, four-plasmid system decreases the likelihood of potential recombination events between the vector genome and the *gag-pol* mRNA that could create RCL particles, though this is still a potential concern in large-scale production systems. Furthermore, a large portion of the HIV-1 3’ U3 enhancer/promoter sequence was deleted in the vector genome to create self-inactivating (SIN) vectors^18^. This prevents transfer of this sequence to the 5’ end during reverse transcription, thereby eliminating the enhancer and promoter elements in the integrated genome and reducing the potential for activating expression of surrounding genes in target cells.

Importantly, vector genome constructs often still contain over 1.5 kilobase (kb) of HIV-1 sequence, including substantial regions of *gag* and *env*, and it is not clear whether all of this is required for transduction and transgene expression. To further decrease potential RCL production and potential interactions between the integrated provirus and host cell genome, vector genomes with Cre/loxP-mediated excision of HIV-1 sequences were designed^19^. However, this introduces the *cre* gene into the genomic RNA and substantially reduces the space available for promoter/transgene cassettes. Another approach was used for the LTR1 vector in which the genome was engineered so that the packaging sequences and RRE are present at the 3’ end of the genomic RNA instead of the 5’ end and are not reverse transcribed, thereby eliminating them from the provirus^20^. This reduced the HIV-1 sequence in the vector genome to only 4.8% of the full-length genome. However, optimal titre required a large intron derived from EF1α to be inserted at the 5’ end of the vector genome, which highlights that little is known about how splicing in the canonical vectors is regulated and how specific splice sites and nuclear export elements modulate vector titre.

The aim of this study was to characterize the functional role of the viral sequences in the lentiviral vector genome and determine which portions could be removed to produce a smaller and potentially safer vector. We found that 850 nt of HIV-1 *gag* and *env* sequence could be removed from the vector genome without compromising transduction efficiency. This reduces the amount of HIV-1 sequence that could potentially recombine with the packaging vector to produce RCL particles. Because the splicing profile of lentiviral vector genomes are poorly characterized, we used Oxford Nanopore sequencing to determine the proportion of vector genomes that are spliced as well as the donor and acceptor sites that are utilized. This analysis showed that deleting 507 nt of *env*, which includes the HIV-1 canonical splice acceptor 7 (SA7), increased the proportion of unspliced genomic RNAs. Finally, we show that deleting most of *gag* can be combined with moving the RRE to 3’ end of the genome so that it is not be reversed transcribed and integrated in the target cell. Overall, removing non-essential HIV-1 sequences from the vector genome may lead to a smaller, safer system that transfers less viral sequence into a patient’s genome.

## Results

### 850 nt of the HIV-1 *gag *and *env* regions can be deleted in the genomic RNA without compromising vector titre

**Figure 1:**
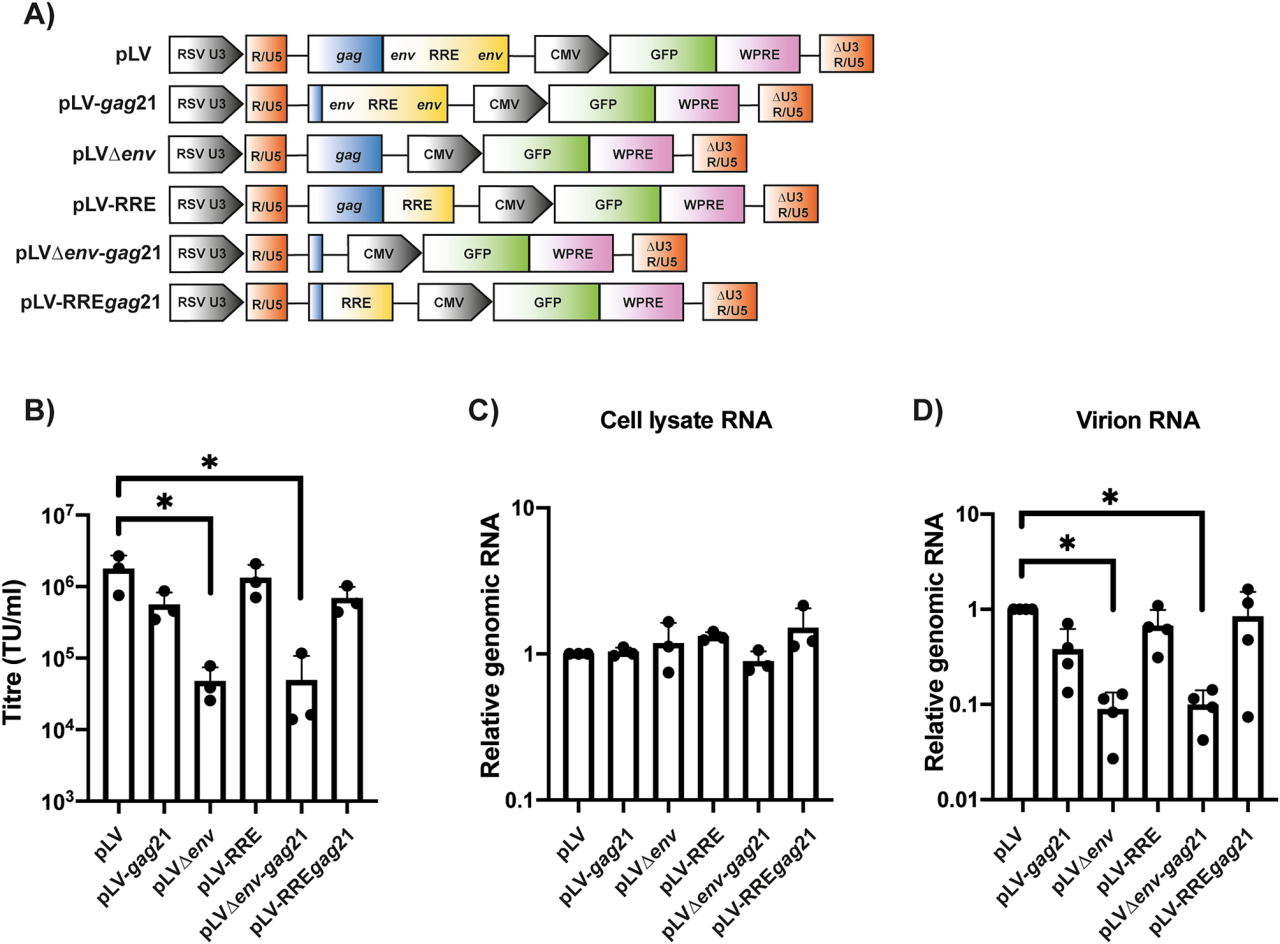
Specific deletions in *gag* and *env* can be made without compromising transduction efficiency. **(A)** Schematic representation of the lentiviral genome vectors. **(B–D)** Lentiviral vectors were produced in HEK293T cells with the indicated genome, packaging construct pCMV∆R8.91 and envelope construct pVSV-G. **(B)** Infectious titres were determined in transduced HEK293T cells by flow cytometry for GFP-positive cells. The bar chart shows the average values of three independent experiments. Data are shown as mean ± SD, *p < 0.05 as determined by a one-way ANOVA test. **(C,D)** Relative genomic RNA abundance within the whole cell lysates **(C)** and virions **(D)** were determined by qRT-PCR. The bar charts show the average values of three **(C)** or 4 **(D)** independent experiments normalized to the value obtained for pLV. Data are shown as mean ± SD, *p < 0.05 as determined by a one-way ANOVA test.

To determine which sequences derived from HIV-1 in the lentiviral vector genome plasmid (pLV) are required for efficient transduction, we analyzed the effect of deleting the *gag* and *env* regions either alone or in combination. Briefly, pLV^21^ contains the Rous sarcoma virus enhancer/promoter^17^, the HIV-1 5’ R-U5-leader region that contains sequences required for genomic RNA dimerization and encapsidation as well as splice donor 1 (SD1)^8,22,23^, a 364 nucleotide (nt) HIV-1 *gag* sequence with a frameshift mutation^8^, an 858 nt HIV-1 *env* sequence^8^ that contains the 351 nt RRE^6,24^ and the splice acceptor used for most fully spliced viral RNAs (SA7)^23^, the HIV-1 central polypurine tract (cPPT) that increases transduction efficiency^25^, a CMV-GFP reporter sequence, the WPRE^10^ and the HIV-1 3’ LTR with a 400-nucleotide deletion that abolished its promoter activity^18^. Sequences within *gag* have been proposed to regulate HIV-1 genomic RNA encapsidation, pre-mRNA splicing, nuclear export and reverse transcription^23,26–29^. Previous studies have reported that deletions in the *gag* region present in lentiviral vectors reduced titre^30–32^, though a 75 nt deletion in the 3’ end of the region was tolerated^33^. However, we have shown that when nt 22–378 in *gag* were deleted in the context of full-length HIV-1, production of infectious VSV-G pseudotyped virus was similar to wild-type HIV-1^34^. While the amino acid sequence within this region is required for HIV-1 Env incorporation due to the interaction between Gag and the C-terminal tail of Env, this is dispensable when a heterologous envelope protein is expressed^35–37^, implying that viral RNA elements in *gag* outside of the dimer structure^38^ are not required for infectious virus production. Therefore, we made pLV-*gag*21, in which 343 nt of the *gag* sequence is removed leaving only the first 21 nt containing a highly conserved sequence that is incorporated into the genomic RNA dimer structure (Fig. 1A)^38–41^. Deleting these 343 nt did not substantially decrease vector titre, intracellular genomic RNA expression or virion-associated genomes (Fig. 1B–D).

To determine the functional relevance of the RNA sequences in the *env* region, we first deleted it (pLV∆*env*, Fig. 1A), which caused an ~ 40-fold decrease in vector titre and ~ tenfold decrease in genomic RNA in virions (Fig. 1B,D). When 343 nt of *gag* and all of *env* were deleted (pLV∆*env*-*gag*21), vector titre and genomic RNA in virions were also substantially reduced. When the 351 nt RRE was inserted in place of *env* to create pLV-RRE or pLV-RRE*gag*21, vector titre and genomic RNA abundance was similar to pLV (Fig. 1B–D). This indicates that while the RRE potently promotes vector titre, other sequences in *env*, including SA7, are not required. This allows 507 nt to be deleted from the *env* region and 343 nt to be deleted from the *gag* region without substantially inhibiting transduction efficiency. In total, this removes 850 nt of HIV-1 sequence from the vector genome.

### The RRE and WPRE are required for optimal vector titre and transgene expression but do not control cytoplasmic genomic RNA abundance

All lentiviral vectors derived from HIV-1 have the major splice donor (SD1) in the 5’ leader region and this sequence is part of the genomic RNA dimer structure^42^. Many lentiviral vectors also have SA7 in the *env* sequence^8^. This leads to the vectors containing a short functional intron. Because intron-containing RNAs are usually retained in the nucleus and targeted for degradation, a nuclear export element is required for optimal cytoplasmic expression of the vector genome^43^. Lentiviral vectors often contain two viral RNA nuclear export elements that could function in the producer cell: the HIV-1 RRE, which interacts with Rev to mediate export through the CRM1 pathway, and the WPRE^6,14,15,24^. However, pLV-RRE does not contain the HIV-1 SA7 sequence and therefore does not have a canonical splice acceptor site. Therefore, we analyzed the effect of deleting the WPRE and the RRE on vector titre and genomic RNA abundance in this vector. To validate our protocol for analyzing cytoplasmic RNA abundance, we used a Rev-dependent subgenomic HIV-1 Gag-Pol-Vif construct^44^ that contains SD1 and the splice acceptor (SA) sites used for *vif* and *vpr* mRNAs, SA1 and SA2. As expected, Gag expression from this vector required Rev (Supplementary Fig. S1A). In the absence of Rev, there was a large decrease in cytoplasmic *gag-pol* RNA abundance and a moderate decrease in nuclear RNA abundance, which reflects decreased intron-containing RNA nuclear export and stability (Supplementary Fig. S1B–D). Therefore, an HIV-1 construct that produces a transcript with one canonical viral splice donor and two splice acceptors requires Rev for cytoplasmic accumulation of the intron-containing RNA.

**Figure 2:**
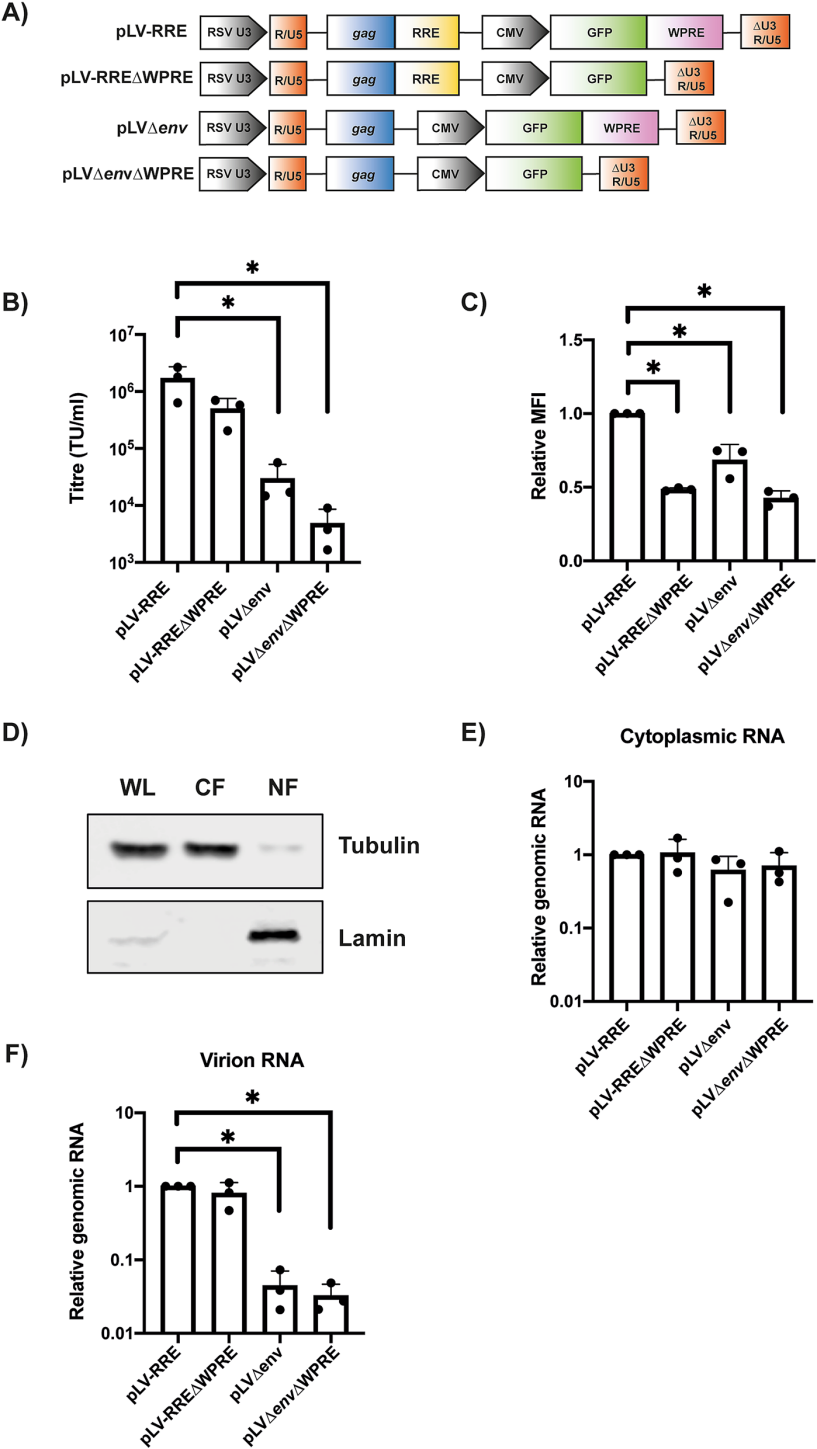
Deletion of the RRE or WPRE reduces transduction efficiency but does not affect cytoplasmic genomic RNA abundance. **(A)** Schematic representation of the lentiviral vector genomes. **(B–E)** Lentiviral vectors were produced in HEK293T cells with the indicated genome, packaging construct pCMV∆R8.91 and envelope construct pVSV-G. **(B)** Infectious titres were determined in transduced HEK293T cells by flow cytometry for GFP-positive cells. The bar chart shows the average values of three independent experiments. Data are shown as mean ± SD, *p < 0.05 as determined by a one-way ANOVA test**. (C)** The relative mean fluorescence intensity (MFI) of GFP-positive cells was determined, indicating the expression efficiency for the transgene. **(D)** Western blotting for the cytoplasmic marker, α-tubulin, and the nuclear marker, lamin-B1, was performed on the HEK293T fractions as a control for fractionation for the whole cell lysate (WL), cytoplasmic fraction (CF) and nuclear fraction (NF) using anti-α-tubulin and anti-lamin-B1 antibodies for the pLV sample. The α-tubulin and lamin-B1 blots are from independent gels. **(E)** Relative genomic RNA abundance within the cytoplasmic fraction was determined by qRT-PCR. **(F)** Relative genomic RNA abundance in the virions was determined by qRT-PCR. **(C,E,F)** The bar charts show the average values of three independent experiments normalized to the value obtained for pLV-RRE. Data are shown as mean ± SD, *p < 0.05 as determined by a one-way ANOVA test.

When the WPRE was deleted in the lentiviral vector (pLV-RRE∆WPRE, Fig. 2A), there was a small, non-significant decrease in vector titre and a decrease in transgene expression in the target cell (Fig. 2B,C). However, there was no decrease in the amount of cytoplasmic or packaged genomic RNA (Fig. 2D–F), suggesting that this element does not contribute to the cytoplasmic accumulation of the viral RNA. Of note, low nuclear RNA levels prevented reproducible analysis of this compartment for this vector. When both the RRE and WPRE were deleted (pLV∆*env*∆WPRE), there was a > 300-fold decrease in vector titre, which correlated with a large decrease in the amount of genomic RNA in virions as well as a decrease in transgene expression in the target cell (Fig. 2B–E). Importantly, there was no significant decrease in cytoplasmic genomic RNA abundance implying that neither the RRE nor WPRE are required to promote cytoplasmic accumulation of this transcript. Interestingly, Rev has been reported to be required for genomic RNA packaging in addition to its role in intron-containing RNA nuclear export^45–47^, and our data is consistent with this additional function for Rev.

### Deleting 507 nt in *env* increases the proportion of unspliced genomic RNAs

**Figure 3:**
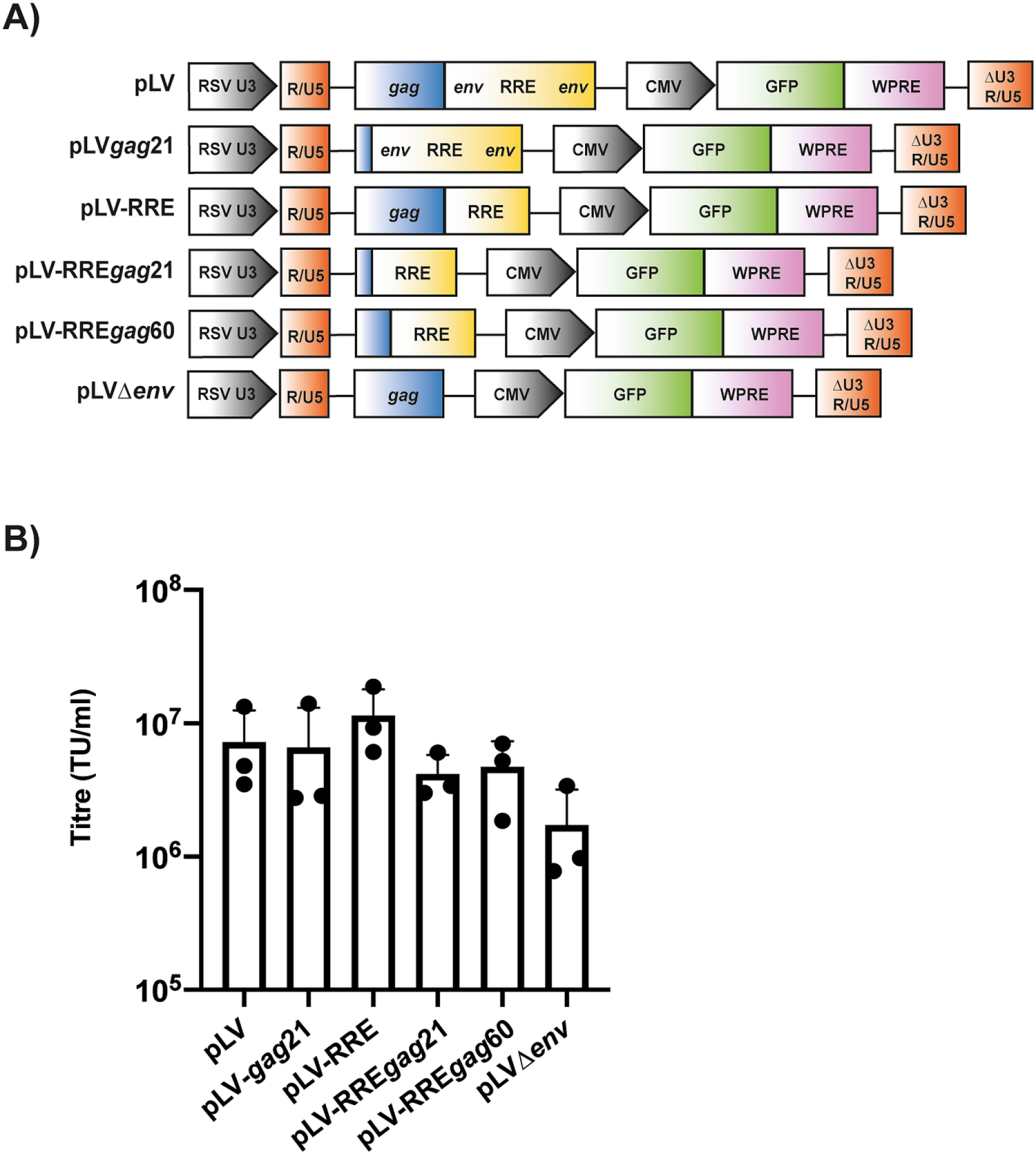
850 nt can be eliminated from the lentiviral vector genome without decreasing titre in HEK293Tsa cells. **(A)** Schematic representation of the lentiviral vector genomes. **(B)** Lentiviral vectors were produced in HEK293Tsa cells with the indicated vector genome, packaging construct pCMV∆R8.91 and envelope construct pVSV-G. Infectious titres were determined in transduced HEK293Tsa cells by digital droplet PCR (ddPCR). The bar charts show the average values of three independent experiments. Data are shown as mean ± SD, A one-way ANOVA test did not identify any samples as p < 0.05.

Next, we analyzed the titre for pLV, pLV-*gag*21, pLV-RRE and pLV-RRE*gag*21 (Fig. 3A-B) using a ddPCR assay in the HEK293Tsa cell line^48^, which produces high titre vectors. All four vectors had similar titres, further indicating that deleting regions in *gag* or *env* did not significantly impair vector production. Deletion of the entire *env* region in the vector genome (pLV∆*env*) decreased titre approximately fourfold. This is a smaller effect size compared to similar experiments in HEK293T cells (Fig. 1D), indicating that the HEK293Tsa cells are less sensitive to alterations in the vector. However, it should be noted that this is a producer cell line used to make clinical-grade lentiviral vectors and most further experiments were performed in this cell line.

**Figure 4:**
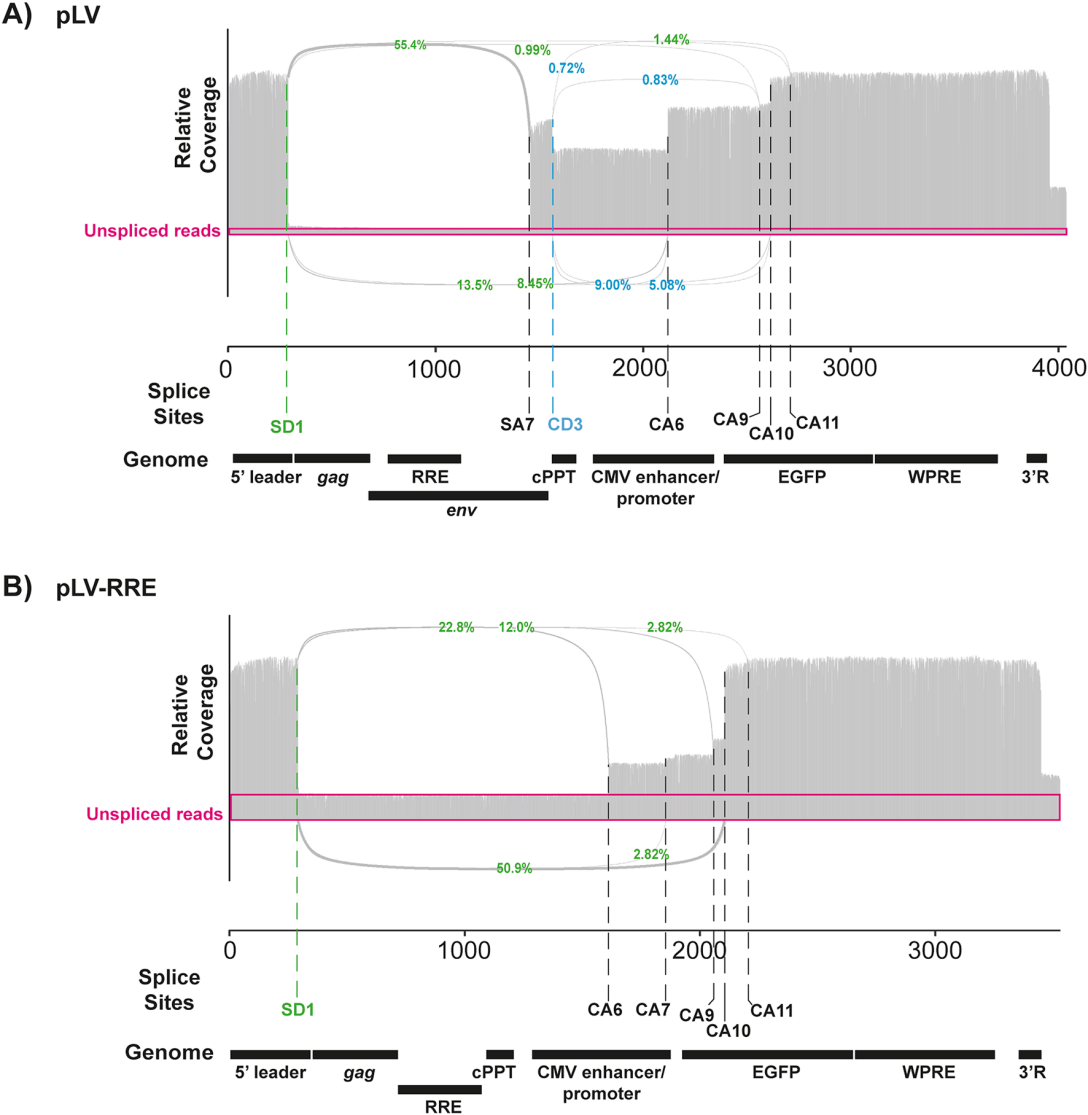
Deletion of 507 nt from the *env* region increases the proportion of unspliced genomic RNA in HEK293Tsa cells. The usage of canonical and cryptic splice donors and acceptors in **(A)** pLV and **(B)** pLV-RRE was determined by Oxford Nanopore sequencing. The percentage of total splicing events supporting the use of splice donor (SD1, green) or cryptic donor 3 (CD3, blue) is shown. The pink boxes represent relative unspliced read coverage. Only junctions supported by at least 10 independent reads are annotated in the sashimi plots. The genomic RNA features for each vector are depicted in the “genome” track.

**Figure 5:**
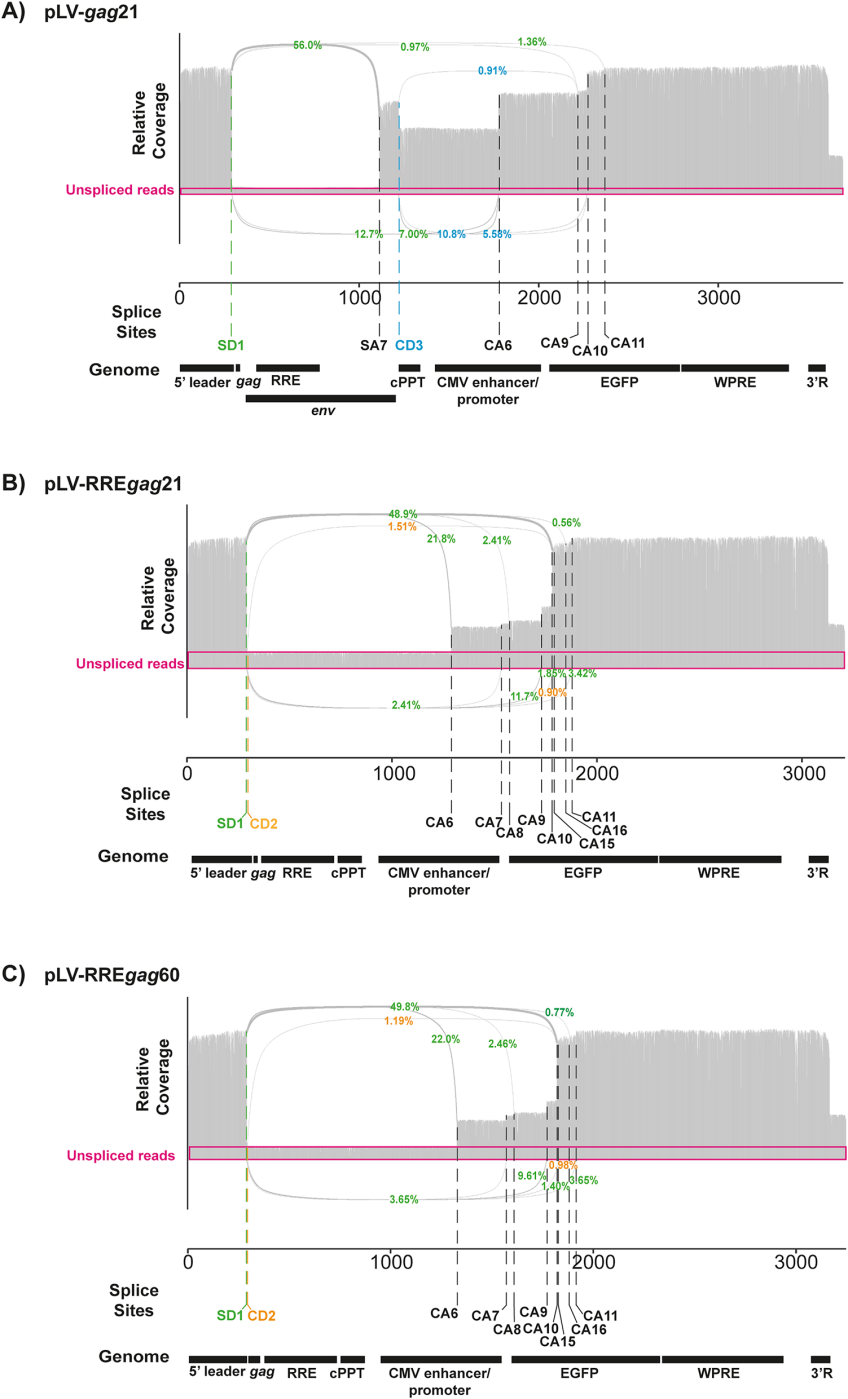
Deletions in *gag* can be combined with the 507nt deletion in env to increase the proportion of unspliced genomic RNA in HEK293Tsa cells. The usage of canonical and cryptic splice donors and acceptors in **(A)** pLV-*gag*21, **(B)** pLV-RRE*gag*21 and **(C)** pLV-RRE*gag*60 was determined using Oxford Nanopore sequencing. The percentage of total splicing events supporting the use of SD1 (green) and CD2 (orange) and CD3 (blue) is shown. The pink boxes represent relative unspliced read coverage. Only junctions supported by a minimum of 10 independent reads are annotated in the sashimi plots. The genomic RNA features for each vector are depicted in the “genome” track.

RNAs transcribed from lentiviral vector genome plasmids can be spliced using SD1 and SA7 as well as cryptic splice donors and acceptors. These events decrease the amount of full-length RNA available for packaging into virions and could lead to packaging of genomes containing internal deletions. Because there are splice sites or splicing enhancer/silencer elements in both *gag* and *env*^23^, we compared how deleting each region affected intracellular genomic RNA splicing in RNA isolated from transfected HEK293Tsa cells. Oxford Nanopore direct cDNA sequencing was used to characterize the splice variants because the long-read length allows full-length transcripts to be analyzed. Even though this sequencing approach initiates at the 3’ end, > 500 near-full length transcripts (defined as reads containing at least one nucleotide upstream of SD1) for each vector were sequenced. These reads were used for further analysis, which allowed a comprehensive characterization of the splicing events and 3’ cleavage site for each vector genome. A substantial number of reads (~ 30%) contained HIV-1 sequence beyond the 3’ R (Figs. 4 and 5, Table 1, Supplementary Table S1), indicating that 3’ end cleavage did not occur at the standard site for HIV-1 transcripts. This has been previously observed and is likely due to elimination of upstream poly(A) regulatory elements by the U3 deletion in the SIN vector^19,49,50^. Of note, there is a SV40 poly(A) signal downstream of the HIV-1 3’ LTR in these vector plasmids, and 3’ cleavage of the readthrough transcripts likely terminates there.

**Table 1:** Splicing events in lentiviral vector genomic RNAs.

	pLV	pLV-RRE	pLV-*gag*21	pLV-RRE*gag*21	pLV-RRE*gag*60
% Unspliced transcripts	4.90	15.3	5.71	12.1	10.2
% Readthrough 3’ R	29%	28%	30%	34%	34%
Splice events/transcript	1.11	0.83	1.13	0.89	0.91
**% of total splicing events**					
SD1-A7	55.4	NA	56.0	NA	NA
SD1-CA6	13.5	22.8	12.7	21.8	22.0
SD1-CA9	0.99	12.0	0.97	11.70	9.61
SD1-CA10	8.45	50.9	6.95	48.9	49.8
CD3-CA6	9.00	NA	10.8	NA	NA
CD3-CA10	5.08	NA	5.58	NA	NA

For pLV, pLV-*gag*21, pLV-RRE and pLV-RRE*gag*21, SD1 and 11 cryptic splice donors (CDs) were used in conjunction with SA7 or 29 cryptic splice acceptors (CAs) (Supplementary Table S1). Importantly, comparing pLV to pLV-RRE showed that deleting 507 nt from *env*, including SA7, decreased the number of splicing events per transcript and increased the proportion of unspliced genomic RNAs approximately threefold in the cell (Fig. 4, Table 1). Splicing events from SD1 to three predominant cryptic splice acceptors (CA6, CA9, CA10) were present in pLV-RRE, including a predominant cryptic acceptor (CA10) in GFP, but these were utilized less frequently than splicing from SD1 to SA7 plus cryptic splice acceptors for pLV. In addition, the internal splicing events from CD3 in pLV, which represent ~ 15% of the total splicing events, are not present in pLV-RRE because this sequence has been deleted. Therefore, deleting 507 nt of *env* decreases the number of splicing events and increases the proportion of unspliced transcripts.

RNA transcribed from pLV and pLV-*gag*21 had similar splicing patterns with many splicing events from SD1 to SA7 as well as splicing events using cryptic donors and acceptors (Figs. 4A and 5A, Table 1). Both vectors had a similar number of splicing events per transcript and proportion of unspliced genomes in the cells (Table 1). However, deleting 507 nt of *env* in the context of the *gag* deletion in pLV-RREgag21 decreased the number of splicing events per transcript and increased the percentage of unspliced transcripts compared to pLV-*gag*21 (Fig. 5A,B, Table 1). This shows that deleting 850 nt of *gag* and *env* does not compromise the vector titre (Fig. 3B) and increases the proportion of unspliced genomic RNAs in cells (Fig. 5B). Because it has previously been reported that a stem-loop structure in nts 26–58 in *gag* helps stabilize the leader region structure for packaging^46^, we characterized a vector with this region of *gag* (pLV-RRE*gag*60). It had a similar titre and splicing pattern to pLV-RRE*gag*21 (Figs. 3B and 5C), which may indicate that another region of the pLV-RRE*gag*21 vector genomic RNA sequence has compensated for it. Overall, deleting 850 nt from the *gag* and *env* regions removes a large portion of the HIV-1 sequence from the lentiviral vector genome without significantly reducing its titre and increases the proportion of unspliced transcripts available for packaging. However, vector titre is not increased in the context of a larger proportion of unspliced transcripts (Fig. 3B), possibly indicating that genome availability is not limiting for infectious virus production under these conditions, or the increase in the proportion of unspliced transcripts is not large enough to substantially increase titre (see Discussion).

### The RRE can be moved to the 3’ end of the genome so that it is not reverse transcribed and combined with the 343 nt deletion in gag without substantially compromising vector titre

**Figure 6:**
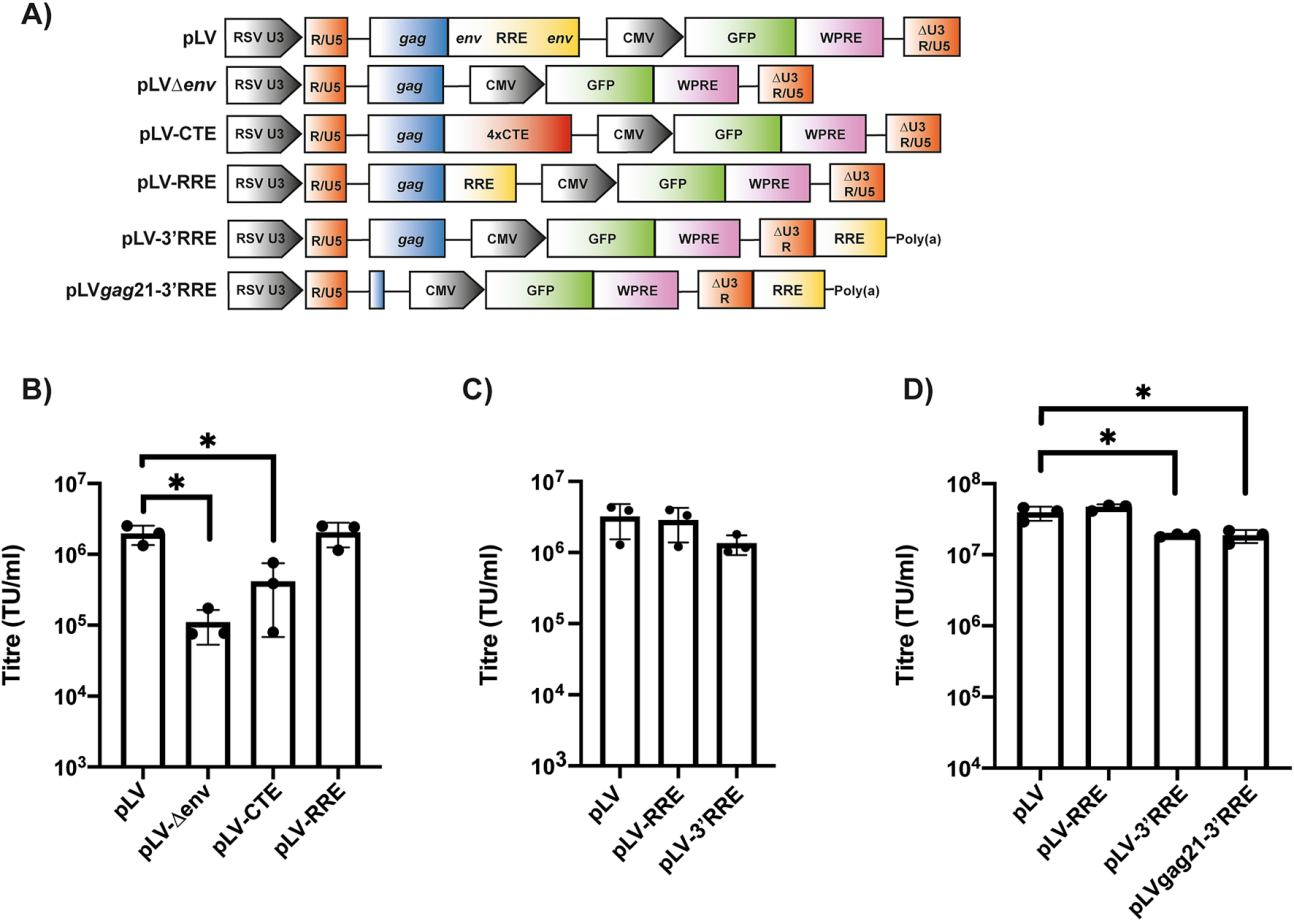
The RRE can be eliminated from the provirus in target cells by moving it downstream of the 3’ R. **(A)** Schematic representation of the lentiviral genome vectors. **(B,C)** Lentiviral vectors were produced in HEK293T cells with the indicated vector genome, packaging construct pCMV∆R8.91 and envelope construct pVSV-G. Infectious titres were determined in transduced HEK293T cells by flow cytometry for GFP + cells. **(D)** Lentiviral vectors were produced in HEK293Tsa cells with the indicated vector genome, packaging construct pCMV∆R8.91 and envelope construct pVSV-G. Infectious titres were determined in transduced HEK293Tsa cells by digital droplet PCR (ddPCR). The bar charts show the average values of three independent experiments. Data are shown as mean ± SD, *p < 0.05 as determined by a one-way ANOVA test.

To determine if the amount of HIV-1 RNA in the vector genome could be further reduced, we replaced the RRE with four copies of the Mason-Pfizer monkey virus (M-PMV) constitutive transport element (CTE)^51–53^ (pLV-4xCTE, Fig. 6A). This retroviral nuclear export element uses the NXF1 nuclear export pathway, which is the route that most cellular mRNA take, instead of the CRM1 pathway used by Rev-dependent HIV-1 intron-containing RNAs^43^. Of note, we have previously shown that four copies of the CTE promotes protein expression in human cells better than one copy^53^. The vector titre for this construct in HEK293T cells was lower than pLV-RRE but higher than pLV∆*env* (Fig. 6B), indicating that the RRE is more efficient for lentiviral vector production than an RNA nuclear export element from a different retrovirus.

We also tested the effect of moving the RRE downstream of the HIV-1 3’ R with the SV40 poly(A) signal in place of U5 (pLV-3’RRE, Fig. 6A). With this modification, the RRE is present in the genomic RNA in the producer cell and virion but is not transferred to the integrated provirus in the target cell as it is excluded from reverse transcription^20^. This reduces the amount of viral sequence present in the transduced cell to potentially reduce interactions between the provirus and the genome of the host cell. It should be noted that this configuration differs from the previously described LTRI vectors^20^ that also have the RRE downstream of the 3’ R in that: (1) pLV-3’RRE has the packaging signal at its standard position at the 5’ end of the genome instead of downstream of the 3’ R; (2) pLV-3’RRE does not contain a heterologous intron, which was required for efficient LTRI vector titre; (3) pLV-3’RRE does not have the 3’ U5 sequence and therefore the SV40 poly(A) sequence is the only complete poly(A) signal. When HEK293T cells were used as the producer cells, pLV-3’RRE had a similar vector titre compared to pLV or pLV-RRE (Fig. 6C), indicating that this configuration did not substantially inhibit infectivity.

Finally, we analyzed whether combining the 343 nt *gag* deletion with moving the RRE downstream of 3’ R was compatible with high titre lentiviral vector production in the HEK293Tsa cell line using a ddPCR assay (Fig. 6D). There is a small (twofold) decrease in titre using this assay when the RRE is moved downstream of 3’ R, though there is still > 1 × 10^7^ TU/ml for pLV-3’RRE without concentration by ultracentrifugation. Importantly, the deletion in *gag* does not further reduce vector titre. Therefore, 850 nt of HIV-1 sequence can be removed from the *gag* and *env* regions and the RRE can be moved downstream of the 3’ R without substantially reducing vector titre. This reduces the HIV-1 sequence present in the provirus after reverse transcription by 1,201 nt.

## Discussion

Herein, we have conducted a systematic analysis of the viral sequences present in a lentiviral vector genome to determine which regions are not essential for transduction efficiency and could potentially be eliminated. There are several HIV-1 sequences in lentiviral vector genomes including the 5’ leader region that regulates dimerization, encapsidation and reverse transcription^22^, a 5’ region of *gag*^8^, the RRE^24^ and splicing signals^23^ embedded within the long stretch of *env* sequence as well as other sequences required for efficient reverse transcription^25,54^. There is also the woodchuck hepatitis virus WPRE in many lentiviral vectors^10^. However, the full functional relevance of many of these sequences for vector transduction efficiency remains unclear.

Most importantly, we found that 850 nt of HIV-1 sequence in the *gag* and *env* regions of the vector genome can be deleted without compromising titre. While there are several reported *cis*-acting RNA elements in these regions, only the RRE is required. Deleting this 850 nt of HIV-1 sequence has several potential advantages. First, it decreases the sequence identity between the genome and packaging vectors that could recombine and produce RCLs. Second, since transduction efficiency decreases as the genome length increases^55^, reducing the HIV-1 portion of the vector genome could increase the capacity for promoters and transgenes. Third, deleting the non-RRE portion of the *env* sequence simplifies the splicing profile of the vector. We also showed that the RRE can be moved distal to the 3’ R region and combined with the large deletion in *gag* without substantially decreasing transduction efficiency. Moving the RRE to this position eliminates it from the vector genome after reverse transcription in target cells, thereby reducing the amount of viral sequence in the integrated provirus^20^.

While the proportion of unspliced RNA was increased up to threefold by removing 507 nt of the *env* region, several splicing events remain, and vector titre was not substantially increased. A possible reason that vector titre did not increase could be that the cytoplasmic abundance of the genomic RNA is in excess of the amount required for efficient packaging. While we did these experiments using small scale three plasmid transfection of HEK293T or HEK293Tsa cells, it will be interesting in the future to compare the titre of the pLV and pLV-RRE genomes in a more clinically relevant setting, such as the recently described bacterial artificial chromosome system^56^, in which the relative abundance of the vector genome and viral proteins may be different. Another possible reason that vector titre did not substantially increase for pLV-RRE compared to pLV is that the threefold increase in the proportion of the unspliced RNA is not large enough to raise the titre. Even in the absence of a canonical HIV-1 splice acceptor, ~ 85% of the vector transcripts are spliced for pLV-RRE. It would be ideal to eliminate all splicing in the genomic RNA since the only relevant transcript for transduction is the unspliced RNA. However, this may be a challenge. While the deletions in *env* to produce pLV-RRE and pLV-RRE*gag*21 eliminate CD3, which contributes to removing an internal portion of the genome in ~ 15% of pLV transcripts, many splicing events that utilize SD1 remain. Several cryptic splice acceptors can be used in the SD1 splicing events, including acceptor sites in the transgene as exemplified by CA10 in the GFP open reading frame. The SD1 sequence is part of the three-way junction in the complex leader RNA structure that mediates dimerization and encapsidation^42^. Therefore, mutations that eliminate SD1 would have to be made in a context that does not affect this structure and cryptic donor sites may be utilized if SD1 is eliminated. As we have shown, Oxford Nanopore sequencing is an excellent tool to determine the complete splicing profile of the genomic RNA due to its long-read length. The specific splicing profile is likely to differ depending on the transgene cassette and should be evaluated in the context of specific therapeutic constructs. Using Oxford Nanopore sequencing or similar technologies may be useful for evaluating the effect of potential cryptic splice sites in transgenes or regulatory elements introduced into the vector genome.

Of note, there are potential safety risks for integrated lentiviral vectors due to interactions between the vector and host cell genomes. Splice sites within the lentiviral vector have been reported to be used in combination with splice sites in cellular genes to produce fusion transcripts between vector and cellular RNAs, which could contribute to vector-mediated genotoxicity^20,57,58^. In particular, splicing between a lentiviral vector transcript and *HMGA2* transcripts resulted in the dysregulation of *HMGA2* expression in a patient treated for β-thalassemia, resulting in benign clonal expansion of hematopoietic progenitors^59^. While we have not evaluated whether eliminating HIV-1 sequences containing cryptic splice sites affects interactions between the vector and cellular transcripts, this is a potential advantage of the minimal pLV*gag*21-3’RRE vector. Therefore, this vector is an alternative to the previously described LTR1 constructs^20^ in that both have the *env* region eliminated from the integrated vector genome but differ in that pLV*gag*21-3’RRE contains the HIV-1 SD1 while LTR1 has a heterologous intron.

Furthermore, in HIV-1 infected patients, interactions between the vector and wild type virus can lead to the vector becoming mobilized by HIV proteins provided in *trans.* This could result in the packaging of vector RNA in HIV-1 particles and spread transduction beyond the intended target cells or to newly infected individuals^60,61^. While the enhancer and promoter elements are eliminated in integrated SIN vectors^18^, mobilization of the integrated vector genome is not totally eliminated^61^ and moving the RRE downstream of the 3’ R reduces the risk of this problem in HIV-1 infected cells^20^. Recombination between the vector genome and wild type viral genome could also occur. Thus, for immunotherapies based on lentiviral vector transduction of CD4 + T cells that may be delivered to HIV-1 positive patients, using lentiviral vectors with substantially reduced amounts of HIV-1 sequence may be beneficial.

Interestingly, while the RRE is essential for nuclear export of HIV-1 intron-containing RNA^24^, the most prominent effect of deleting this region in the context of the pLV-RRE vector is on the amount of genomic RNA packaged into virions. Therefore, in the context of a genome that does not have multiple canonical HIV-1 splice sites, the RRE appears to be more important for packaging the genome into virions than promoting cytoplasmic RNA abundance. Because pLV-RRE only contains one canonical splice donor (SD1) in contrast to HIV-1, which contains at least four defined splice donors and eight splice acceptors^23^, its RNA may have a lower requirement for Rev-RRE-mediated nuclear export than the full-length virus. It also does not contain many of the cis-acting repressive sequences (CRSs)/instability sequences (INSs) found in HIV-1 RNAs that require Rev for efficient expression^24^. Furthermore, the WPRE has been reported to be an RNA nuclear export element^13–15^, which could make it functionally redundant with the RRE. However, deletion of the RRE, WPRE or both elements together did not substantially affect cytoplasmic genomic RNA abundance, indicating that they are not required for nuclear export of this transcript. The hepatitis B virus post-transcriptional regulatory element and WPRE have recently been shown to promote gene expression by recruiting the TENT4–ZCCHC14 complex^16^. This leads to a ‘mixed tail’ at the 3’ end of mRNAs, in which non-adenosine nucleotides are inserted into the poly(A) tail, that protects the mRNA from degradation and may be the mechanism by which the WPRE promotes gene expression from lentiviral vector genomes. Rev and the RRE have previously been reported to be required for efficient genomic RNA encapsidation^45–47^ and our data supports those observations. The specific mechanism by which Rev and the RRE regulate HIV-1 or lentiviral vector genomic RNA packaging remains unclear and is an important area for further investigation.

In summary, we have shown that it is possible to decrease the length of lentiviral vector genomes by deleting non-essential regions in *gag* and *env* and moving the RRE downstream of 3’ R, which eliminates it from the integrated provirus. This may improve vector design by increasing transgene capacity, reducing potential RCL formation and decreasing the amount of splicing that occurs in the vector transcripts. This could also potentially reduce genotoxicity by decreasing the number of fusion transcripts created by splicing between the integrated vector and cellular RNAs, though this would have to be validated in the context of a therapeutic model system. While we used a GFP transgene in the experiments in this report, analyzing long, complex transgenes such as next generation CAR or T cell receptor (TCR) constructs in the pLV*gag*21-3’RRE construct would help determine its clinical potential. These transgene cassettes express proteins in addition to the CAR or TCR to improve T cell expansion and persistence in vivo, render transduced T cells resistant to the immunosuppressive tumor microenvironment or enable clinical intervention to selectively eliminate transduced cells in vivo if severe toxicity occurs^62,63^. The size and complexity of these and other therapeutic transgenes that can be delivered using lentiviral vectors may benefit from a minimal vector genome with decreased splicing potential. Overall, removing non-essential HIV-1 elements from lentiviral vector genomes may enhance vector performance and reduce the amount of viral sequence transduced into the genome of a patient’s cells during gene therapy.

## Materials and methods

### Plasmids

The previously described lentiviral vector genome constructs used in this study was pLV (pRRL-PPT-CMV-GFP-WPRE)^21^. The *gag* and *env* deletions were made in pLV using overlapping PCR. The WPRE was removed by cutting at EcoRI sites flanking the WPRE sequence. The 4xCTE sequence transferred from pGPV-4xCTE^53^ using the NotI and EcoRI sites into pLV∆*env* to create pLV-CTE. For pLV-3’RRE, the RRE and SV40 poly(A) sequences were synthesized using GenScript fragment synthesis and inserted into the KpnI and AvrII sites in pLV∆*env*. pCMV∆R8.91^9^, pGag-Pol-Vif (pGPV)^44^, pVSV-G^64^, pRev^53^ and pGFP^65^ have previously been described.

### Cells

HEK293T and HEK293Tsa^48^ cells were grown in Dulbecco’s Modified Eagle Medium (DMEM) plus Gluta-Max (Life Technologies) supplemented with 10% FBS and 1% penicillin–streptomycin. Cells were maintained in a humidified atmosphere with 5% CO_2_ at 37 °C.

### Vector production

HEK293T or HEK293Tsa cells were seeded 24 h prior to transfection at a density of 10^6^ cells per well in a six-well plate. Plasmids for transfection was prepared in Opti-MEM. Each well was co-transfected with 1 µg vector genome, 1 µg packaging plasmid and 0.5 µg pVSV-G using PEI at a DNA/PEI ratio of 1:3. Media was changed 6 h post-transfection and the supernatant containing viral vector particles were filtered through a 0.45 μm Millex-HA filter (Millipore) 48 h post-transfection.

### Quantification of vector titre by FACS

HEK293T or HEK293Tsa were plated in 96-well plates as target cells for transduction. A serial dilution of supernatant containing viral vectors was prepared and added to the wells. Cells were harvested 48-h post-transduction, fixed in 2% paraformaldehyde, and re-suspended in 1 × phosphate-buffered saline (PBS). Samples were run on a flow cytometer to detect GFP positive cells which was used to calculate the titer (transducing units (TU)/ml).

### Quantitative RT-PCR

Cells were washed with 1xPBS and the RNA was extracted using the RNeasy mini kit (Qiagen) following the manufacturer’s instructions. To extract virion RNA, the supernatant was centrifuged at 20,000×*g* for 2 h through a 20% sucrose cushion in 1 × PBS to pellet the vector particles. Virion RNA was extracted using the QIAamp Viral mini kit (Qiagen). 1 μg of cellular RNA and 20 µL of virion RNA was reverse transcribed using the High Capacity cDNA Reverse-Transcription kit (Applied Biosystems). Quantitative PCR was performed using the Taqman Universal PCR mix and the QuantiStudio 5 System (Thermo Fisher). Absolute quantification was determined using a standard curve of the lentiviral vector DNA plasmid. The genomic RNA primers were TCTCGACGCAGGACTCG/TACTGACGCTCTCGCACC (forward/reverse), and the probe was FAM-ATCTCTCTCCTTCTAGCCTC-TAMRA. Of note, this primer–probe set spans SD1 and only detects unspliced genomic RNA.

### RNA isolation

A protocol previously optimized to analyze HIV-1 cytoplasmic abundance was used to isolate cytoplasmic and nuclear viral transcripts^44^. Briefly, 48 h post transfection, the cells were lysed on ice in 400 µL of cold, NB buffer (50 mM Tris- HCL pH 8.0, 20 mM NaCl, 1.5 mM MgCl_2_, 0.5% NP-40), and centrifuged at 500×*g* for 10 min to pellet nuclei. 200 µL of the cytoplasmic supernatant was added to 600 µL of RLT buffer (Qiagen). The nuclear pellet was washed twice in cold NB buffer, resuspended in 400 µL of RLT buffer (Qiagen) and spun through a Qiashredder column (Qiagen). The cytoplasmic and nuclear RNA was extracted using the RNeasy Mini kit (Qiagen).

### Western Blotting

48 h post-transfection, whole cell or the cytoplasmic and nuclear fractions were lysed in radioimmunoprecipitation (RIPA) buffer (10 mM Tris–HCl, pH 7.5, 150 mM NaCl, 1 mM EDTA, 0.1% SDS, 1% Triton X-100, 1% sodium deoxycholate) and 2 × loading buffer (60 mM Tris–HCl (pH 6.8), 10% β-mercaptoethanol, 10% glycerol, 2% sodium dodecyl sulfate (SDS), 0.1% bromophenol blue). Cell lysates were resolved by SDS–polyacrylamide gel electrophoresis and transferred to a nitrocellulose membrane. Protein bands were detected using the LI-COR infrared imaging system (LI-COR UK LTD). The antibodies used were 1:50 HIV- 1 anti-p24^Gag^ (183-H12-5C)^66^, 1:1000 anti-Hsp90 (Santa Cruz Biotechnology, sc7947), 1:5000 anti-α-Tubulin antibody (Abcam, ab7291), 1:1000 anti-Lamin B1 (Abcam, ab8982), 1:10,000 Dylight™ 800-conjugated anti-mouse/rabbit secondary antibodies (Cell Signalling Technology). The Rev-specific monoclonal antibody Rev-6 (1:500) was provided by Michael Malim and was generated by immunizing BALB/c mice with purified hexahistidine-tagged HIV-1_HXB3_ Rev, boosting three times and recovering the spleen to generate hybridomas with the mouse plasmacytoma cell line SP2/0Ag. Hybridoma culture supernatants were screened by enzyme linked immunosorbent assay for reactivity against plastic-adhered antigen.

### Quantification of vector titre by Digital Droplet PCR

HEK293Tsa cells^48^ were transduced with 50, 5 and 0.5 µL of supernatant containing viral vectors. 72 h post-transduction, cells were harvested, and genomic DNA was isolated using the DNeasy Blood and Tissue kit (Qiagen). Approximately 50 ng of extracted genomic DNA was digested using MluI-HF (New England Biolabs). From the digestion mixture, 5 µL of digested genomic DNA was added to a PCR reaction mix containing 2 × ddPCR Supermix for Probes (no dUTP) (Bio-Rad), a primer/probe set for the RNaseP reference gene (TaqMan Copy Number Reference Assay, Applied Biosystems) and a primer/probe set for the target, which spans the SD1 and therefore specifically detects unspliced genomic RNA (TCTCGACGCAGGACTCG/CGCTCTCGCACCCATCTC (forward/reverse) and probe FAM-CTCCTTCTAGCCTCCGCTAG-BHQ1), at a final concentration of 0.9 μM of each primer and 0.25 μM of each probe. 20 µL of the final mix was added to a 96-well plate, and droplets were generated using the Automated Droplet Generator (Bio-Rad) following the manufacturer’s instructions. PCR was performed using the C1000 Touch Thermal Cycler (Bio-Rad) and droplets were read using a QX200 droplet reader. The average copy number per cell was used to calculate titre (TU/ml).

### Analysis of lentiviral vector splicing using Oxford Nanopore sequencing

HEK239Tsa cells^48^ were transfected with pLV, pLV-*gag*21, pLV-RRE, pLV-RRE*gag*21 or pLV-RRE*gag*60, along with pCMV∆R8.91 and pVSV-G. 48 h post-transfection, RNA was extracted using the RNeasy Mini kit (Qiagen). Polyadenylated mRNA was isolated from approximately 10 µg of total RNA using the Dynabeads mRNA purification kit (ThermoFisher Scientific) following the manufacturer’s instructions. The quality and quantity of mRNA was assessed using the TapeStation 4200 (Agilent). Nanopore libraries were prepared with 100 ng of poly(A) + RNA using the Direct cDNA Sequencing kit (SQK-DCS109) with Native Barcoding (EXP-NB104) following the manufacturer’s instructions (Oxford Nanopore Technologies). The final library was loaded onto an R9.4.1 MinION flow cell (FLO-MIN111, Oxford Nanopore Technologies) and sequenced for 72 h.

The barcoded raw sequence data (FAST5 files) generated by Oxford Nanopore sequencing were basecalled and debarcoded using GUPPY basecaller version 3.1.5 to output decoded FASTQ files. The debarcoded FASTQ files for each vector were selected using samtools version 1.10. The reads were then aligned to the human genome (hg38) and the lentiviral vector genome simultaneously using minimap2^67^. Near-full length vector genome reads (reads that contain at least 1 nucleotide upstream of SD1) were selected using samtools. Lentiviral vector-mapping junction-spanning reads were isolated using regtools (https://github.com/griffithlab/regtools) to allow per-junction read counting. The percentage of HIV-1 junction-spanning reads was calculated by dividing the number of reads for each junction by the total number of junction-spanning reads in the library. Sashimi plots were generated using ggsashimi^68^.

### Statistical analysis

Statistical significance was determined using a one-way ANOVA test. Data are represented as mean ± SD. Significance was ascribed to p values p < 0.05.

### Ethics declarations

No human tissue samples, human subjects or animals were used in this study.

## Supplementary Information

Supplementary Information.

## Data Availability

The Oxford Nanopore RNA-seq datasets are available at GSE165829.
