# Supplementary material for: HIV-1 sequences in lentiviral vector genomes can be substantially reduced without compromising transduction efficiency
Source: Sci Rep. 2021 Jun 8;11:12067. doi: 10.1038/s41598-021-91309-w (PMC8187449; doi:10.1038/s41598-021-91309-w)
Supplement: Supplementary file 1 — Supplementary Information. [file 41598_2021_91309_MOESM1_ESM.pdf]

HIV-1 sequences in lentiviral vector genomes can be substantially reduced without compromising transduction efficiency

Helin Sertkaya[1], Mattia Ficarelli[1], Nathan P Sweeney[2], Hannah Parker[1], Conrad A Vink[2*], Chad M Swanson[1*]

[1]Department of Infectious Diseases, King's College London, London, SE1 9RT, UK

[2]Cell & Gene Therapy Platform, Medicinal Science and Technology, GSK, Stevenage, SG1 2NY, UK

*Corresponding authors: Conrad A Vink (conrad.x.vink@gsk.com) and Chad M Swanson (chad.swanson@kcl.ac.uk)

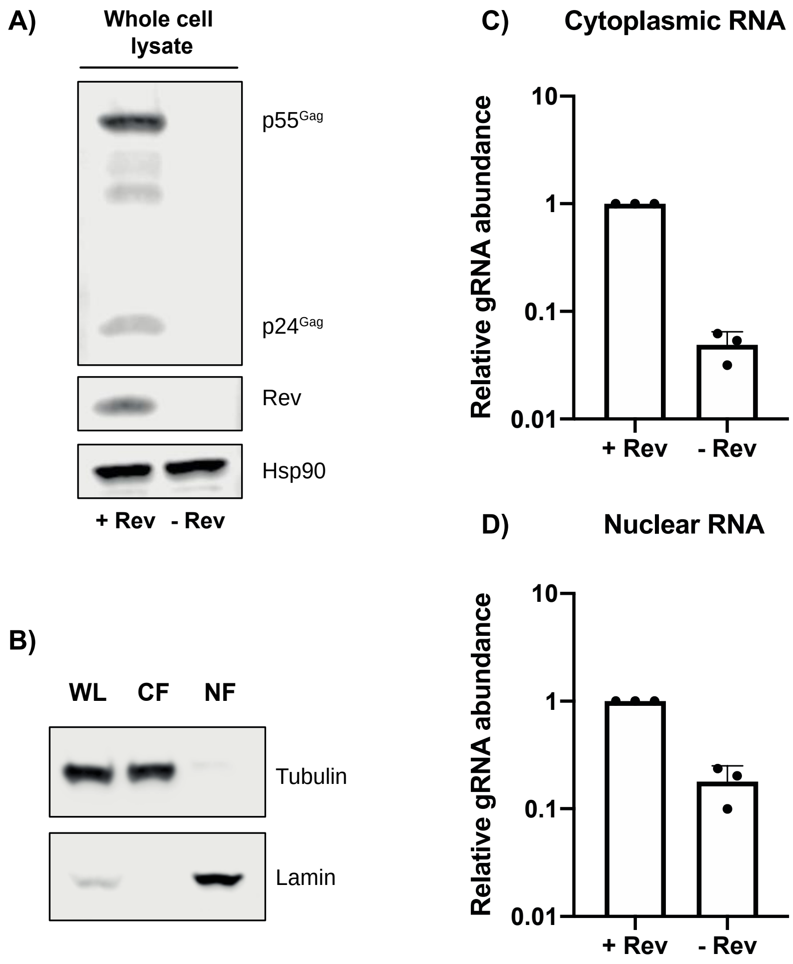

Figure S1: Rev is essential for the nuclear RNA export of a subgenomic HIV-1 construct

HEK293T cells were transfected with a subgenomic HIV-1 construct expressing Gag, Pol and Vif (pGPV) plus pRev or pGFP. **(A)** Intracellular Gag, Rev and HSP90 was determined by western blotting. The Gag blot was independent from the Rev and Hsp90 blots, which were from the same gel. **(B)** The whole lysate (WL) or, after subcellular fractionation, the cytoplasmic fraction (CF) and nuclear fraction (NF) for cells transfected with pGPV plus Rev were western blotted for the cytoplasmic protein, alpha-tubulin, and the nuclear protein, lamin-B1. The alpha-tubulin and lamin-B1 blots are from independent gels. **(C-D)** Relative genomic RNA abundance within the cytoplasmic fraction **(C)** and nuclear fraction **(D)** was determined by qPCR. The bar chart shows the average values of three independent experiments. Data are shown as mean ± SD.

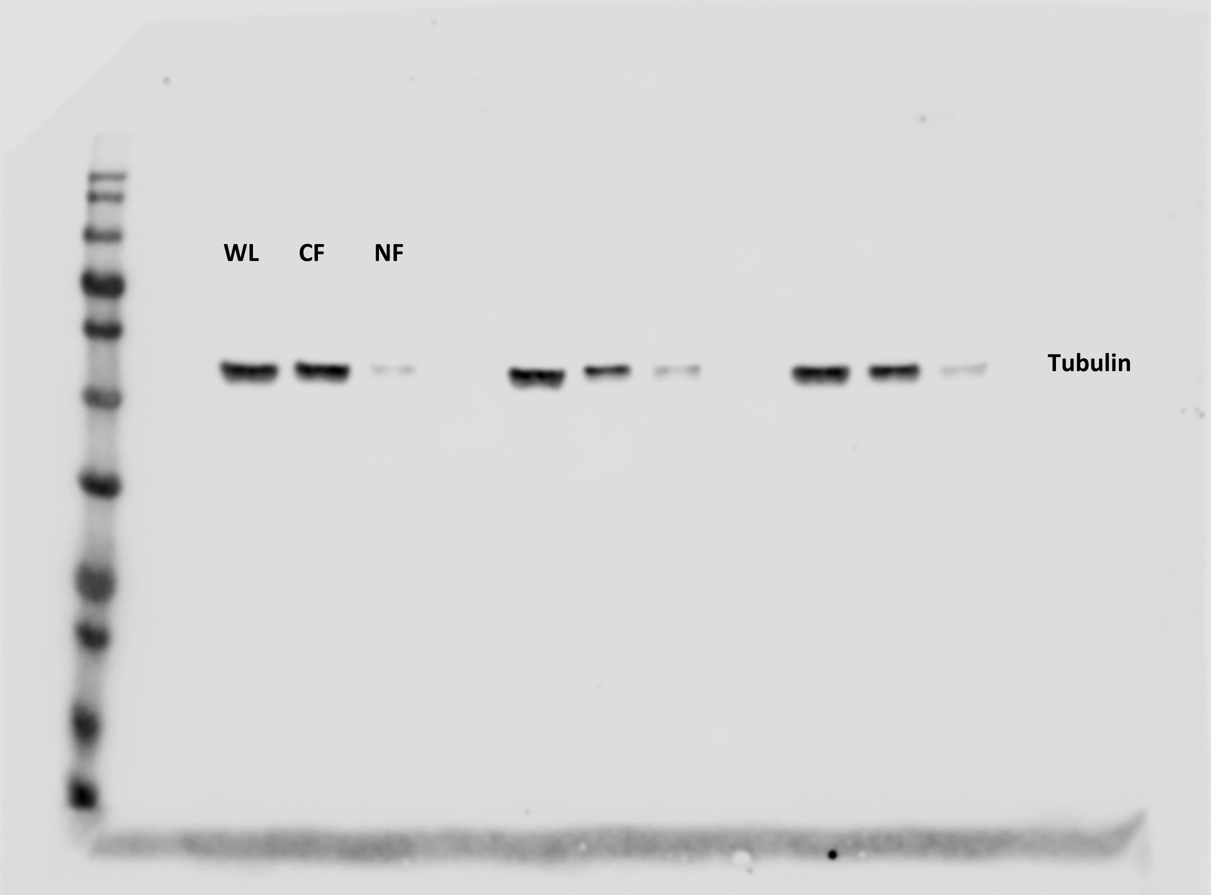

Full blot from Figure 2D

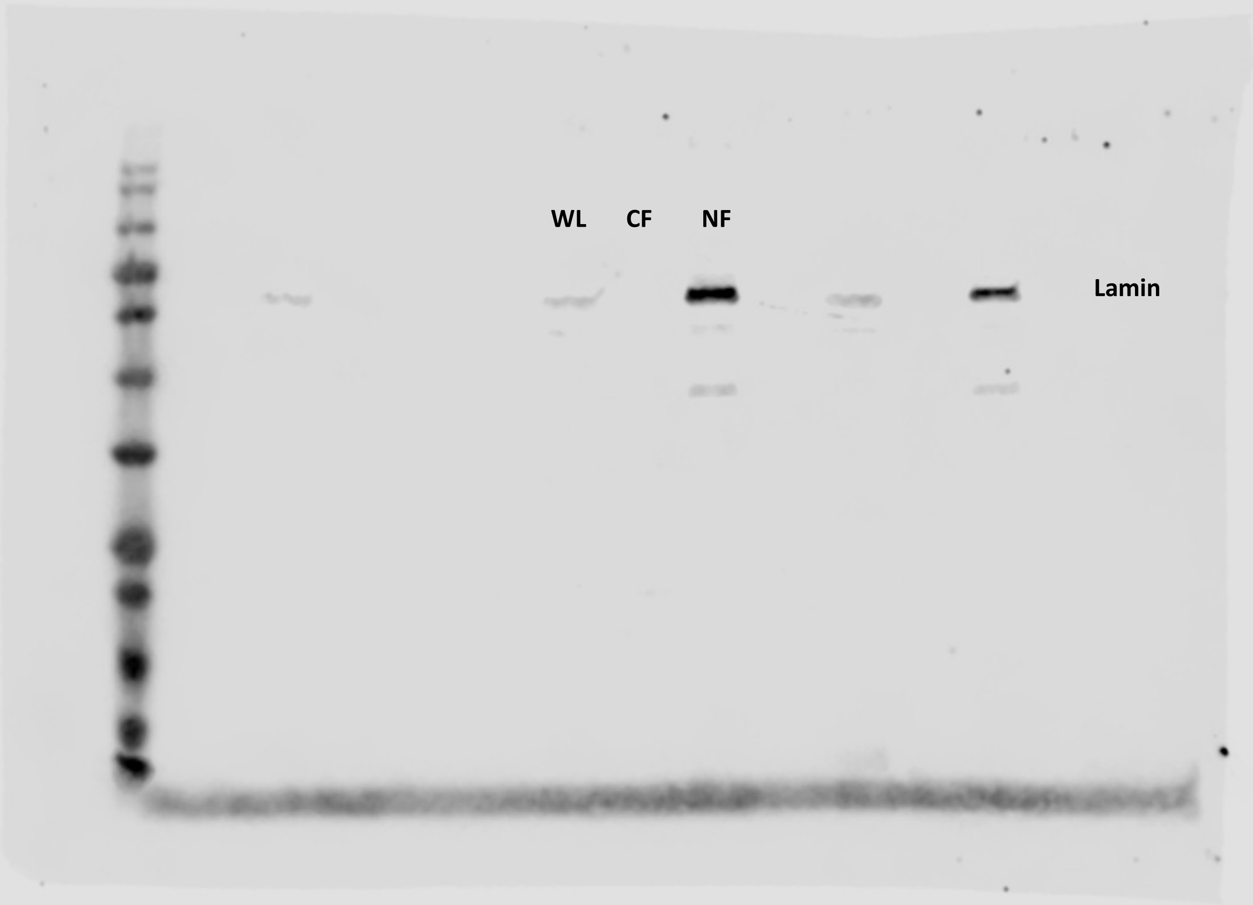

Full blot from Figure 2D

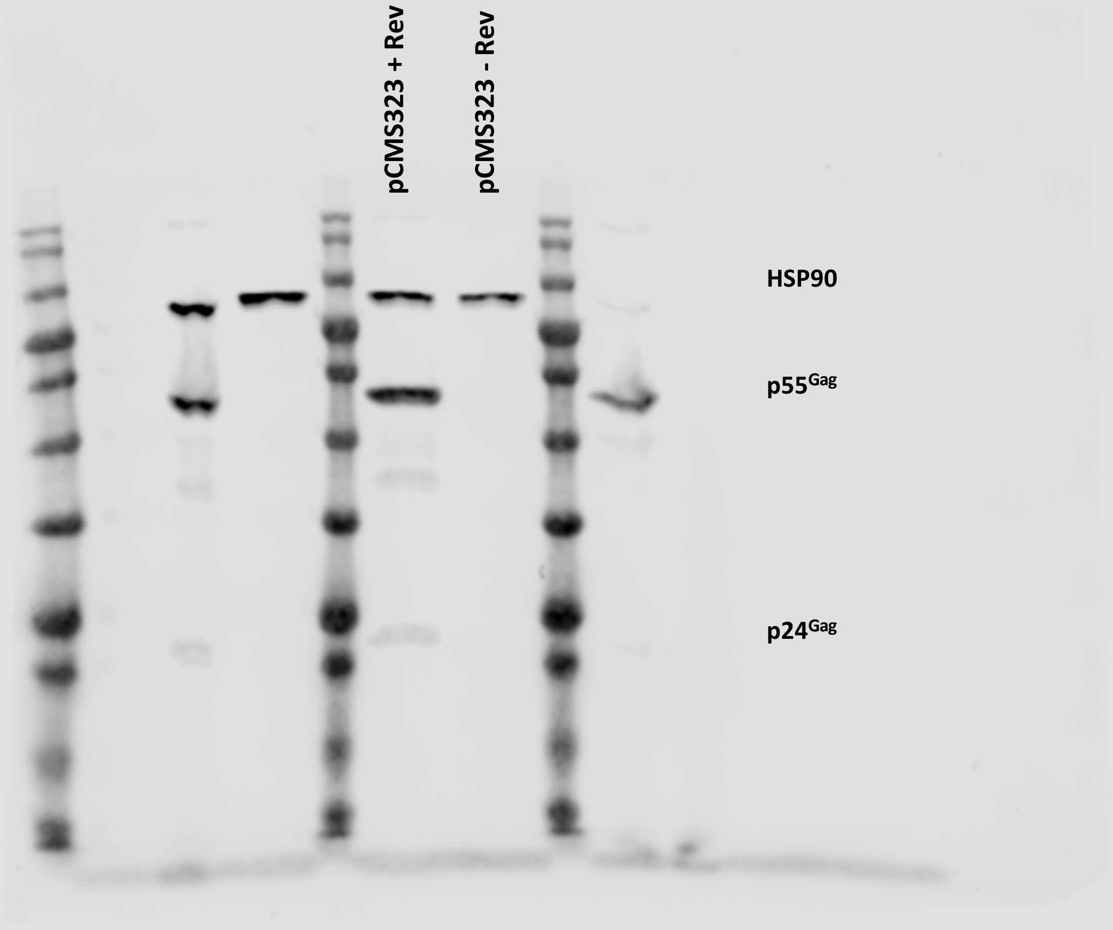

Full blot from Supplementary Figure S1A

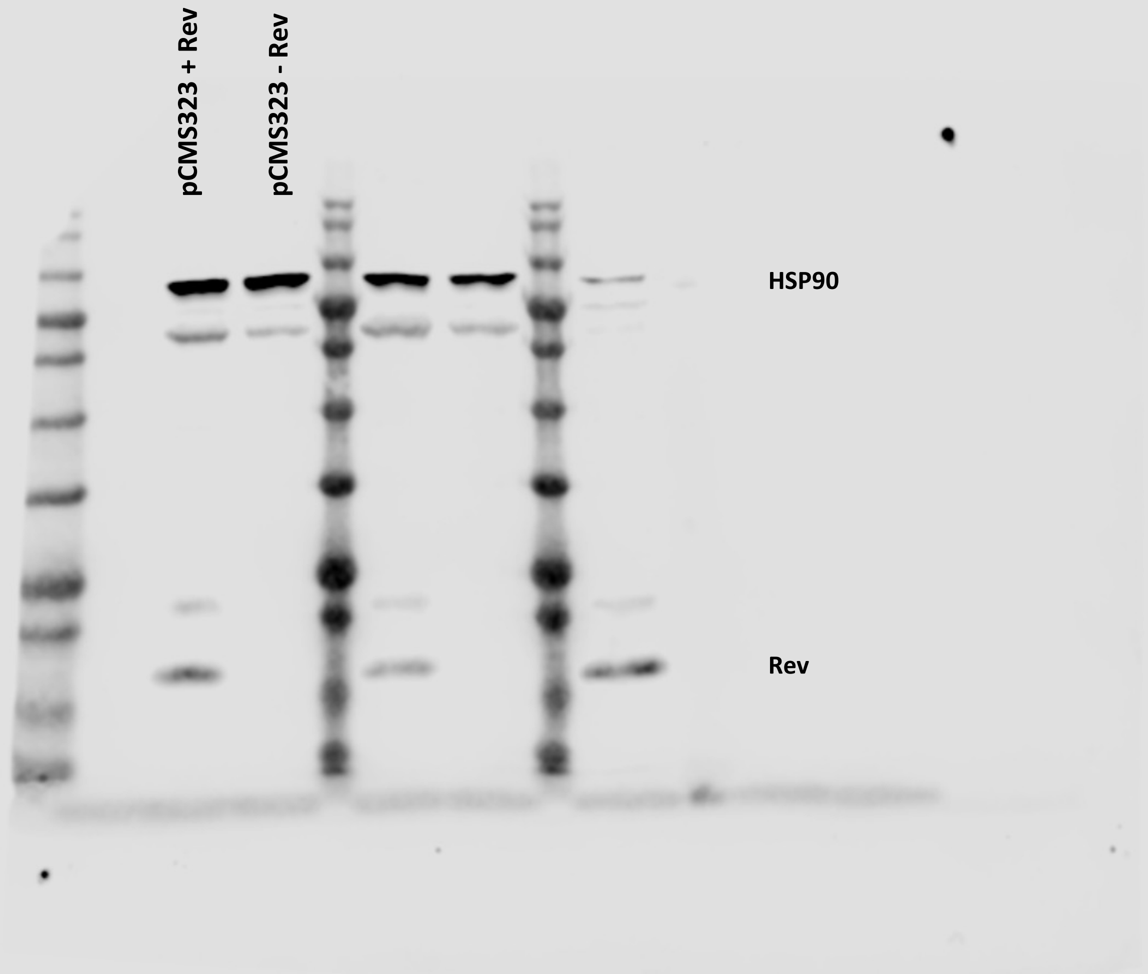

Full blot from Supplementary Figure S1A

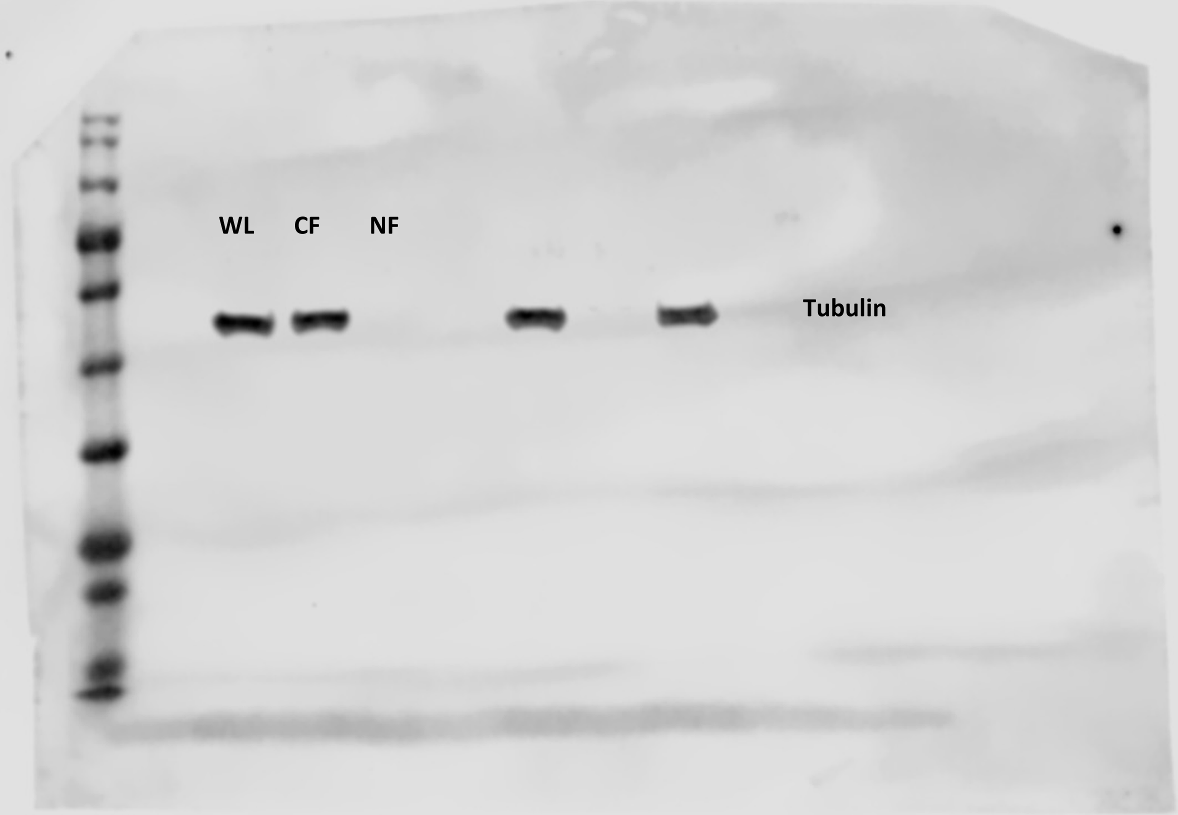

Full blot from Supplementary Figure S1B

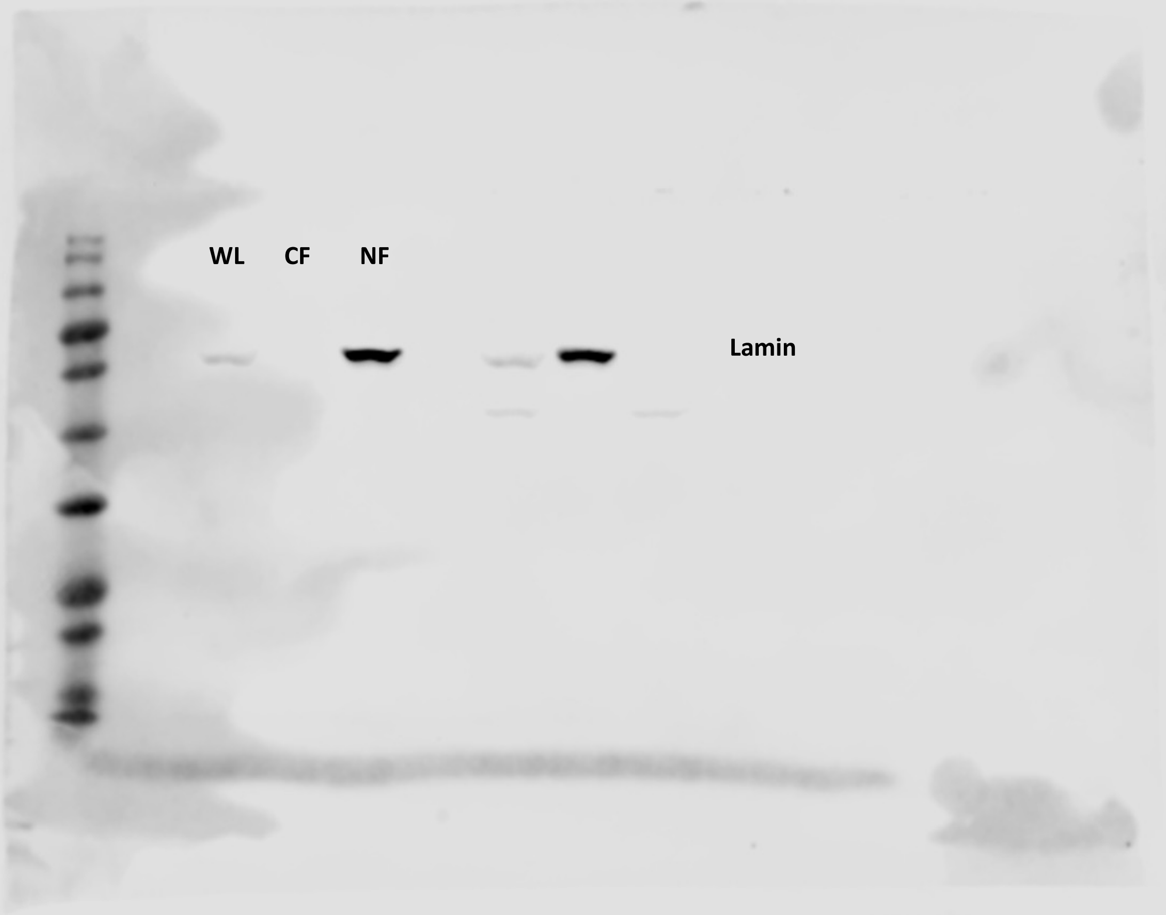

Full blot from Supplementary Figure S1B

Supplementary Table S1

Oxford Nanopore sequencing results for **A)** pLV, **B)** pLV-RRE, **C)** pLV-*gag*21, **D)** pLV-RRE*gag*21, **E)** pLV-RRE*gag*60. The left panel indicates all the detected splice events. Junctions with one read are highlighted in orange, and junctions with more than one read are highlighted in green. The middle panel shows the start and end site of all junctions with more than one read, and each of these junctions are displayed as a percentage of the total splice events. The right panel indicates the total number of spliced and unspliced reads, the total splice events, and the number of readthrough transcripts that read beyond the 3’ R.

A) pLV

Vector	Unamed Junction	Transformed Junc. Start	Transformed Junc. End	Total No. Events
pLV	JUNC00000002	291	2612	153
pLV	JUNC00000198	131	2708	1
pLV	JUNC00000001	291	1456	1003
pLV	JUNC00000057	131	242	5
pLV	JUNC00000007	291	2708	26
pLV	JUNC00000004	291	2119	244
pLV	JUNC00000096	195	1613	1
pLV	JUNC00000016	291	2561	18
pLV	JUNC00000006	291	1823	2
pLV	JUNC00000103	247	3129	1
pLV	JUNC00000111	295	1488	5
pLV	JUNC00000171	291	2642	1
pLV	JUNC00000211	255	3242	1
pLV	JUNC00000088	291	611	2
pLV	JUNC00000020	295	1456	5
pLV	JUNC00000200	291	1010	1
pLV	JUNC00000021	291	1165	1
pLV	JUNC00000083	291	1488	3
pLV	JUNC00000206	291	2401	1
pLV	JUNC00000029	291	2363	8
pLV	JUNC00000095	291	1476	3
pLV	JUNC00000240	382	1095	1
pLV	JUNC00000233	398	1370	1
pLV	JUNC00000076	453	3725	1
pLV	JUNC00000154	632	1098	1
pLV	JUNC00000127	649	3939	1
pLV	JUNC00000028	637	3739	1
pLV	JUNC00000037	679	3638	1
pLV	JUNC00000069	688	3738	1
pLV	JUNC00000213	1180	2579	1
pLV	JUNC00000003	1563	2119	163
pLV	JUNC00000026	1563	2708	13
pLV	JUNC00000008	1563	2612	92
pLV	JUNC00000098	1582	3147	1
pLV	JUNC00000010	1563	2561	15
pLV	JUNC00000195	1563	2401	2
pLV	JUNC00000074	1690	2015	1
pLV	JUNC00000058	1672	3144	1
pLV	JUNC00000236	1693	3773	1
pLV	JUNC00000045	2064	3714	1
pLV	JUNC00000032	2169	2256	1
pLV	JUNC00000052	2198	3092	1
pLV	JUNC00000208	2183	3618	1
pLV	JUNC00000100	2589	3010	1
pLV	JUNC00000238	2595	2998	1
pLV	JUNC00000068	2708	3119	1
pLV	JUNC00000190	2728	2812	1
pLV	JUNC00000094	2838	3129	1
pLV	JUNC00000160	2830	2996	1
pLV	JUNC00000087	2871	3089	1
pLV	JUNC00000036	2878	2999	1
pLV	JUNC00000073	2905	3453	1
pLV	JUNC00000185	2904	3584	1
pLV	JUNC00000161	3010	3091	1
pLV	JUNC00000081	3006	3341	1
pLV	JUNC00000162	3005	3641	1
pLV	JUNC00000181	3065	3527	1
pLV	JUNC00000135	3044	3618	1
pLV	JUNC00000199	3163	3880	1
pLV	JUNC00000203	3112	3246	1
pLV	JUNC00000117	3171	3568	1
pLV	JUNC00000149	3238	3542	1
pLV	JUNC00000102	3185	3560	1
pLV	JUNC00000059	3213	3638	1
pLV	JUNC00000151	3464	3631	1
pLV	JUNC00000121	3629	3715	1
pLV	JUNC00000097	3686	4008	1

start	end	splice_sites	events	% of total events
131	242	CD1-CA1	5	**0.28**
291	611	SD1-CA2	2	**0.11**
291	1456	SD1-A7	1003	**55.38**
291	1476	SD1-CA3	3	**0.17**
291	1488	SD1-CA4	3	**0.17**
291	1823	SD1-CA5	2	**0.11**
291	2119	SD1-CA6	244	**13.47**
291	2363	SD1-CA7	8	**0.44**
291	2561	SD1-CA9	18	**0.99**
291	2612	SD1-CA10	153	**8.45**
291	2708	SD1-CA11	26	**1.44**
295	1488	CD2-CA4	5	**0.28**
295	1456	CD2-A7	5	**0.28**
1563	2119	CD3-CA6	163	**9.00**
1563	2401	CD3-CA8	2	**0.11**
1563	2561	CD3-CA9	15	**0.83**
1563	2612	CD3-CA10	92	**5.08**
1563	2708	CD3-CA11	13	**0.72**

Total near full length transcripts (containing sequence from SD1 to the end of 3' R)	Total splice events.	Total unspliced transcripts	Reads ending at the end of 3' R	Readthrough Reads
1633	1811	80	1159	474

B) pLV-RRE

Vector	Unamed Junction	Transformed Junc. Start	Transformed Junc. End	Total No. Events
pLV-RRE	JUNC00000004	291	1612	97
pLV-RRE	JUNC00000003	291	2105	217
pLV-RRE	JUNC00000028	218	2283	1
pLV-RRE	JUNC00000002	291	2054	51
pLV-RRE	JUNC00000008	291	1894	9
pLV-RRE	JUNC00000012	291	1856	12
pLV-RRE	JUNC00000009	291	2201	12
pLV-RRE	JUNC00000016	291	2063	1
pLV-RRE	JUNC00000010	295	2113	10
pLV-RRE	JUNC00000046	295	2105	3
pLV-RRE	JUNC00000044	291	2270	1
pLV-RRE	JUNC00000043	291	2219	1
pLV-RRE	JUNC00000006	291	2113	6
pLV-RRE	JUNC00000053	291	2059	1
pLV-RRE	JUNC00000005	398	484	1
pLV-RRE	JUNC00000022	2204	2294	1
pLV-RRE	JUNC00000038	2403	2487	1
pLV-RRE	JUNC00000015	2678	3050	1

start	end	splice_sites	events	% of total events
291	1612	SD1-CA6	97	**22.77**
291	1856	SD1-CA7	12	**2.82**
291	1894	SD1-CA8	9	**2.11**
291	2054	SD1-CA9	51	**11.97**
291	2105	SD1-CA10	217	**50.94**
291	2113	SD1-CA15	6	**1.41**
291	2201	SD1-CA11	12	**2.82**
295	2105	CD2-CA10	3	**0.70**
295	2113	CD2-CA15	10	**2.35**

Total near full length transcripts (containing sequence from SD1 to the end of 3' R)	Total splice events.	Total unspliced transcripts	Reads ending at the end of 3' R	Readthrough Reads
515	426	79	373	142

C) pLV-*gag*21

Vector	Unamed Junction	Transformed Junc. Start	Transformed Junc. End	Total No. Events
pLV-gag21	JUNC00000050	131	242	6
pLV-gag21	JUNC00000008	291	2269	107
pLV-gag21	JUNC00000009	291	1776	195
pLV-gag21	JUNC00000013	75	2748	1
pLV-gag21	JUNC00000160	70	3437	1
pLV-gag21	JUNC00000063	70	2767	1
pLV-gag21	JUNC00000001	291	1113	862
pLV-gag21	JUNC00000020	213	1145	1
pLV-gag21	JUNC00000016	163	3510	1
pLV-gag21	JUNC00000019	291	2218	15
pLV-gag21	JUNC00000108	212	1270	1
pLV-gag21	JUNC00000110	212	1808	1
pLV-gag21	JUNC00000005	291	2365	21
pLV-gag21	JUNC00000120	225	2455	1
pLV-gag21	JUNC00000074	274	1113	1
pLV-gag21	JUNC00000011	291	822	5
pLV-gag21	JUNC00000188	291	2302	1
pLV-gag21	JUNC00000124	291	1085	2
pLV-gag21	JUNC00000064	291	2333	2
pLV-gag21	JUNC00000048	291	1125	2
pLV-gag21	JUNC00000015	291	1480	2
pLV-gag21	JUNC00000072	291	2020	4
pLV-gag21	JUNC00000032	295	2277	3
pLV-gag21	JUNC00000190	420	2938	1
pLV-gag21	JUNC00000165	869	2852	1
pLV-gag21	JUNC00000082	998	1259	1
pLV-gag21	JUNC00000088	941	3313	1
pLV-gag21	JUNC00000031	1220	1772	1
pLV-gag21	JUNC00000104	1168	1776	1
pLV-gag21	JUNC00000003	1220	1776	167
pLV-gag21	JUNC00000004	1220	2269	86
pLV-gag21	JUNC00000006	1220	2218	14
pLV-gag21	JUNC00000030	1220	2365	6
pLV-gag21	JUNC00000077	1178	3275	1
pLV-gag21	JUNC00000094	1220	1792	1
pLV-gag21	JUNC00000142	1329	2801	1
pLV-gag21	JUNC00000041	1528	3371	1
pLV-gag21	JUNC00000149	1526	2230	1
pLV-gag21	JUNC00000014	1547	1641	1
pLV-gag21	JUNC00000012	2031	2101	1
pLV-gag21	JUNC00000100	2077	3184	1
pLV-gag21	JUNC00000028	2352	2825	1
pLV-gag21	JUNC00000021	2365	2455	3
pLV-gag21	JUNC00000083	2368	2458	2
pLV-gag21	JUNC00000103	2509	3323	1
pLV-gag21	JUNC00000026	2564	2654	1
pLV-gag21	JUNC00000096	2620	2741	1
pLV-gag21	JUNC00000128	2667	2767	1
pLV-gag21	JUNC00000102	2684	3035	1
pLV-gag21	JUNC00000071	2719	2944	1
pLV-gag21	JUNC00000049	2742	2855	1
pLV-gag21	JUNC00000024	2828	3184	3
pLV-gag21	JUNC00000105	2828	3137	1

start	end	splice_sites	events	% of total events
131	242	CD1-CA1	6	**0.39**
291	822	SD1-CA12	5	**0.32**
291	1085	SD1-CA13	2	**0.13**
291	1113	SD1-A7	862	**55.97**
291	1125	SD1-CA14	2	**0.13**
291	1480	SD1-CA5	2	**0.13**
291	1776	SD1-CA6	195	**12.66**
291	2020	SD1-CA7	4	**0.26**
291	2218	SD1-CA9	15	**0.97**
291	2269	SD1-CA10	107	**6.95**
291	2333	SD1-CA16	2	**0.13**
291	2365	SD1-CA11	21	**1.36**
295	2277	CD2-CA15	3	**0.19**
1220	1776	CD3-CA6	167	**10.84**
1220	2218	CD3-CA9	14	**0.91**
1220	2269	CD3-CA10	86	**5.58**
1220	2365	CD3-CA11	6	**0.39**
2365	2455	CD4-CA17	3	**0.19**
2368	2458	CD5-CA18	2	**0.13**

Total near full length transcripts (containing sequence from SD1 to the end of 3' R)	Total splice events.	Total unspliced transcripts	Reads ending at the end of 3' R	Readthrough Reads
1366	1540	78	951	415

D) pLV-RRE*gag*21

Vector	Unamed Junction	Transformed Junc. Start	Transformed Junc. End	Total No. Events
pLV-RREgag21	JUNC00000046	81	1835	1
pLV-RREgag21	JUNC00000093	131	2347	1
pLV-RREgag21	JUNC00000116	65	2388	1
pLV-RREgag21	JUNC00000032	131	242	1
pLV-RREgag21	JUNC00000006	291	1731	209
pLV-RREgag21	JUNC00000003	291	1782	873
pLV-RREgag21	JUNC00000004	291	1289	390
pLV-RREgag21	JUNC00000001	291	1571	43
pLV-RREgag21	JUNC00000153	250	2459	1
pLV-RREgag21	JUNC00000122	208	2333	1
pLV-RREgag21	JUNC00000031	291	1846	10
pLV-RREgag21	JUNC00000138	214	1968	1
pLV-RREgag21	JUNC00000039	228	1815	1
pLV-RREgag21	JUNC00000025	291	1790	33
pLV-RREgag21	JUNC00000178	223	1571	1
pLV-RREgag21	JUNC00000060	291	1544	3
pLV-RREgag21	JUNC00000002	291	1878	61
pLV-RREgag21	JUNC00000015	291	1533	43
pLV-RREgag21	JUNC00000136	260	2650	1
pLV-RREgag21	JUNC00000007	295	1782	27
pLV-RREgag21	JUNC00000016	295	1790	16
pLV-RREgag21	JUNC00000050	291	1735	2
pLV-RREgag21	JUNC00000144	291	1830	3
pLV-RREgag21	JUNC00000017	291	1812	1
pLV-RREgag21	JUNC00000082	295	1289	7
pLV-RREgag21	JUNC00000092	291	1815	4
pLV-RREgag21	JUNC00000148	291	1875	1
pLV-RREgag21	JUNC00000112	291	1726	1
pLV-RREgag21	JUNC00000012	295	1731	2
pLV-RREgag21	JUNC00000177	383	691	1
pLV-RREgag21	JUNC00000107	405	680	2
pLV-RREgag21	JUNC00000097	754	2073	1
pLV-RREgag21	JUNC00000133	839	2317	1
pLV-RREgag21	JUNC00000096	933	2730	1
pLV-RREgag21	JUNC00000101	1881	1971	3
pLV-RREgag21	JUNC00000034	1878	2001	1
pLV-RREgag21	JUNC00000029	1878	1968	1
pLV-RREgag21	JUNC00000043	1878	1971	1
pLV-RREgag21	JUNC00000022	2073	3129	1
pLV-RREgag21	JUNC00000045	2011	2164	2
pLV-RREgag21	JUNC00000145	1932	2642	1
pLV-RREgag21	JUNC00000142	2027	2707	1
pLV-RREgag21	JUNC00000161	2036	2297	1
pLV-RREgag21	JUNC00000130	2043	2289	1
pLV-RREgag21	JUNC00000147	2006	2141	1
pLV-RREgag21	JUNC00000051	2029	2709	1
pLV-RREgag21	JUNC00000103	2079	2289	1
pLV-RREgag21	JUNC00000072	2075	2731	1
pLV-RREgag21	JUNC00000126	2169	2950	1
pLV-RREgag21	JUNC00000077	2070	2241	1
pLV-RREgag21	JUNC00000157	2074	2187	1
pLV-RREgag21	JUNC00000124	2198	2856	1
pLV-RREgag21	JUNC00000098	2085	2174	1
pLV-RREgag21	JUNC00000080	2173	2733	1
pLV-RREgag21	JUNC00000038	2100	2187	2
pLV-RREgag21	JUNC00000030	2124	2788	1
pLV-RREgag21	JUNC00000019	2124	2568	1
pLV-RREgag21	JUNC00000011	2167	2259	1
pLV-RREgag21	JUNC00000115	2341	2697	2
pLV-RREgag21	JUNC00000061	2217	2788	2
pLV-RREgag21	JUNC00000069	2193	3050	1
pLV-RREgag21	JUNC00000175	2202	2298	1
pLV-RREgag21	JUNC00000104	2282	2462	1
pLV-RREgag21	JUNC00000086	2361	2630	1
pLV-RREgag21	JUNC00000074	2355	2714	1
pLV-RREgag21	JUNC00000099	2405	2812	1
pLV-RREgag21	JUNC00000068	2456	2893	1
pLV-RREgag21	JUNC00000162	2780	2853	1
pLV-RREgag21	JUNC00000168	2860	2950	1

start	end	splice_sites	events	% of total events
291	1289	SD1-CA6	390	**21.84**
291	1533	SD1-CA7	43	**2.41**
291	1544	SD1-CA20	3	**0.17**
291	1571	SD1-CA8	43	**2.41**
291	1731	SD1-CA9	209	**11.70**
291	1735	SD1-CA21	2	**0.11**
291	1782	SD1-CA10	873	**48.88**
291	1790	SD1-CA15	33	**1.85**
291	1815	SD1-CA22	4	**0.22**
291	1830	SD1-CA23	3	**0.17**
291	1846	SD1-CA16	10	**0.56**
291	1878	SD1-CA11	61	**3.42**
295	1289	CD2-CA6	7	**0.39**
295	1731	CD2-CA9	2	**0.11**
295	1782	CD2-CA10	27	**1.51**
295	1790	CD2-CA15	16	**0.90**
405	680	CD6-CA19	2	**0.11**
1881	1971	CD5-CA18	3	**0.17**
2011	2164	CD7-CA24	2	**0.11**
2100	2187	CD8-CA25	2	**0.11**
2217	2788	CD9-CA26	2	**0.11**
2341	2697	CD10-CA27	2	**0.11**

Total near full length transcripts (containing sequence from SD1 to the end of 3' R)	Total splice events.	Total unspliced transcripts	Reads ending at the end of 3' R	Readthrough Reads
2006	1786	243	1330	676

E) pLV-RRE*gag*60

Vector	Unamed Junction	Transformed Junc. Start	Transformed Junc. End	Total No. Events
pLV-RREgag60	JUNC00000046	107	2196	1
pLV-RREgag60	JUNC00000006	291	1917	52
pLV-RREgag60	JUNC00000150	238	1930	1
pLV-RREgag60	JUNC00000003	291	1821	710
pLV-RREgag60	JUNC00000008	291	1770	137
pLV-RREgag60	JUNC00000001	291	1328	314
pLV-RREgag60	JUNC00000002	291	1610	35
pLV-RREgag60	JUNC00000094	213	2328	1
pLV-RREgag60	JUNC00000013	291	1572	52
pLV-RREgag60	JUNC00000110	228	1947	1
pLV-RREgag60	JUNC00000037	244	2304	1
pLV-RREgag60	JUNC00000011	291	1829	20
pLV-RREgag60	JUNC00000166	267	771	1
pLV-RREgag60	JUNC00000149	236	2017	1
pLV-RREgag60	JUNC00000164	237	2872	1
pLV-RREgag60	JUNC00000082	278	1829	1
pLV-RREgag60	JUNC00000042	291	1885	11
pLV-RREgag60	JUNC00000138	291	1854	1
pLV-RREgag60	JUNC00000045	276	1917	1
pLV-RREgag60	JUNC00000077	291	1782	1
pLV-RREgag60	JUNC00000029	295	1829	14
pLV-RREgag60	JUNC00000048	295	1821	17
pLV-RREgag60	JUNC00000036	291	1379	2
pLV-RREgag60	JUNC00000051	291	1692	1
pLV-RREgag60	JUNC00000146	291	1583	3
pLV-RREgag60	JUNC00000127	291	1779	1
pLV-RREgag60	JUNC00000108	313	3153	1
pLV-RREgag60	JUNC00000071	384	725	1
pLV-RREgag60	JUNC00000118	378	719	1
pLV-RREgag60	JUNC00000165	414	2210	1
pLV-RREgag60	JUNC00000055	499	574	1
pLV-RREgag60	JUNC00000081	598	750	1
pLV-RREgag60	JUNC00000101	636	752	1
pLV-RREgag60	JUNC00000104	778	1785	1
pLV-RREgag60	JUNC00000012	1073	1195	1
pLV-RREgag60	JUNC00000016	1469	2340	1
pLV-RREgag60	JUNC00000041	1502	2286	1
pLV-RREgag60	JUNC00000005	1558	2034	1
pLV-RREgag60	JUNC00000014	1683	1796	1
pLV-RREgag60	JUNC00000109	1856	1997	1
pLV-RREgag60	JUNC00000078	1920	2010	1
pLV-RREgag60	JUNC00000033	1915	2007	1
pLV-RREgag60	JUNC00000124	1917	2007	1
pLV-RREgag60	JUNC00000073	2050	2203	3
pLV-RREgag60	JUNC00000049	2054	2189	1
pLV-RREgag60	JUNC00000062	2061	2208	1
pLV-RREgag60	JUNC00000063	2225	2809	1
pLV-RREgag60	JUNC00000088	2048	2201	1
pLV-RREgag60	JUNC00000123	2039	2354	1
pLV-RREgag60	JUNC00000050	2039	2280	1
pLV-RREgag60	JUNC00000145	2044	2353	1
pLV-RREgag60	JUNC00000038	2082	2319	1
pLV-RREgag60	JUNC00000113	2112	2328	1
pLV-RREgag60	JUNC00000031	2113	2226	1
pLV-RREgag60	JUNC00000111	2232	2319	2
pLV-RREgag60	JUNC00000060	2170	2607	1
pLV-RREgag60	JUNC00000141	2209	2328	1
pLV-RREgag60	JUNC00000021	2225	2359	1
pLV-RREgag60	JUNC00000072	2227	2925	1
pLV-RREgag60	JUNC00000052	2256	2827	1
pLV-RREgag60	JUNC00000135	2327	2404	1
pLV-RREgag60	JUNC00000142	2349	2774	1
pLV-RREgag60	JUNC00000134	2380	2689	1
pLV-RREgag60	JUNC00000122	2444	2755	1
pLV-RREgag60	JUNC00000105	2413	2774	1
pLV-RREgag60	JUNC00000028	2503	2607	1
pLV-RREgag60	JUNC00000130	2495	2765	1

start	end	splice_sites	events	% of total events
291	1328	SD1-CA6	314	**22.04**
291	1379	SD1-CA28	2	**0.14**
291	1572	SD1-CA7	52	**3.65**
291	1583	SD1-CA20	3	**0.21**
291	1610	SD1-CA8	35	**2.46**
291	1770	SD1-CA9	137	**9.61**
291	1821	SD1-CA10	710	**49.82**
291	1829	SD1-CA15	20	**1.40**
291	1885	SD1-CA16	11	**0.77**
291	1917	SD1-CA11	52	**3.65**
295	1821	CD2-CA10	17	**1.19**
295	1829	CD2-CA15	14	**0.98**
2050	2203	CD7-CD24	3	**0.21**
2232	2319	CD11-CA29	2	**0.14**

Total near full length transcripts (containing sequence from SD1 to the end of 3' R)	Total splice events.	Total unspliced transcripts	Reads ending at the end of 3' R	Readthrough Reads
1572	1425	160	1032	540
